# Genomic Landscape of Experimental Bladder Cancer in Rodents and Its Application to Human Bladder Cancer: Gene Amplification and Potential Overexpression of Cyp2a5/CYP2A6 Are Associated with the Invasive Phenotype

**DOI:** 10.1371/journal.pone.0167374

**Published:** 2016-11-30

**Authors:** Kazuhiro Kanemoto, Katsuhiro Fukuta, Noriyasu Kawai, Keiichi Tozawa, Masako Ochiai, Koji Okamoto, Sumiko Ohnami, Hiromi Sakamoto, Teruhiko Yoshida, Yae Kanai, Masaru Katoh, Takahiro Yasui, Kenjiro Kohri, Tadao Kakizoe, Hitoshi Nakagama

**Affiliations:** 1 Division of Cancer Development System, National Cancer Center Research Institute, Tokyo, Japan; 2 Department of Nephro-Urology, Nagoya City University Graduate School of Medical Sciences, Nagoya, Japan; 3 Division of Cancer Differentiation, National Cancer Center Research Institute, Tokyo, Japan; 4 Division of Genetics, National Cancer Center Research Institute, Tokyo, Japan; 5 Division of Molecular Pathology, National Cancer Center Research Institute, Tokyo, Japan; 6 Department of Omics Network, National Cancer Center Research Institute, Tokyo, Japan; 7 National Cancer Center Research Institute, Tokyo, Japan; Centro Nacional de Investigaciones Oncologicas, SPAIN

## Abstract

Non-muscle invasive (superficial) bladder cancer is a low-grade malignancy with good prognosis, while muscle invasive (invasive) bladder cancer is a high-grade malignancy with poor prognosis. *N*-butyl-*N*-(4-hydroxybutyl)nitrosamine (BBN) induces superficial bladder cancers with papillary morphology in rats and invasive bladder cancers with infiltrating phenotype in mice. In this study, we analyzed genomic landscapes of rodent BBN-induced bladder cancers using array-based comparative genomic hybridization (array CGH). While no significant copy number alterations were detected in superficial bladder tumors in rats, copy number gains in chromosomal regions 2D-E1, 7qA3, 9F2, and 11C-D were detected in invasive bladder tumors in mice. Amplification of representative genes located on 2D-E1 and 7qA3 chromosomal regions was confirmed by quantitative PCR. *Cyp2a22* and *Cyp2a5* genes but not *Cyp2g1*, *Cyp2a12*, and *Rab4b* genes on mouse chromosome 7qA3 were amplified in invasive bladder cancers. Although the human ortholog gene of *Cyp2a22* has not been confirmed, the mouse *Cyp2a5* gene is the ortholog of the human *CYP2A6* gene located in chromosomal region 19q13.2, and *CYP2A6* was identified by database search as one of the closest human homolog to mouse *Cyp2a22*. Considering a possibility that this region may be related to mouse 7qA3, we analyzed *CYP2A6* copy number and expression in human bladder cancer using cell lines and resected tumor specimens. Although only one of eight cell lines showed more than one copy increase of the *CYP2A6* gene, *CYP2A6* amplification was detected in six out of 18 primary bladder tumors where it was associated with the invasive phenotype. Immunohistochemical analyses of 118 primary bladder tumors revealed that CYP2A6 protein expression was also higher in invasive tumors, especially in those of the scattered type. Together, these findings indicate that the amplification and overexpression of the *CYP2A6* gene are characteristic of human bladder cancers with increased malignancy and that *CYP2A6* can be a candidate prognostic biomarker in this type of cancer.

## Introduction

Human cancers develop because of genetic and epigenetic changes induced by environmental and hereditary factors [1, 2]. Bladder cancer is one of several types of tumors arising in the urinary tract [3–6]. A study involving 44,788 pairs of twins in northern European countries revealed that environmental and hereditary factors contribute to 69% and 31% of the risk for bladder cancer, respectively; however, the data do not reach statistical significance [7], which is probably due to insufficient power of distinguishing between hereditary and environmental effects [8]. Sampson *et al*. have recently reported the estimation of the heritability of bladder cancer and its proportion attributable to cigarette smoking based on genome-wide association studies (GWASs) [9]. Cigarette smoking as well as occupational exposure to paint components, polycyclic aromatic hydrocarbons, and aromatic amines are known as environmental risk factors of bladder cancer [10, 11]. Using candidate-gene approach, a previous study has demonstrated the association between an increased risk of bladder cancer and deletion of the *GSTM1* gene on human chromosome 1p13.3, which is involved in the metabolism of carcinogens [12]. Many GWASs revealed a correlation between an increased risk of bladder cancer and single nucleotide polymorphisms (SNPs) [13–18].

The *FGFR3*, *HRAS*, *ERBB2*, *CCND1*, *MDM2*, and *E2F3* genes are established oncogenes, while *CDKN2A*, *TP53*, *RB1*, *PTEN*, and *PTCH* represent tumor suppressor genes shown to be involved in bladder cancer [5]. Several studies have revealed copy number aberrations in bladder cancer using array-based comparative genomic hybridization (array CGH) [19–22]. In bladder cancer, such genes as *SETDB1* on human chromosomal region 1q21, *FGFR1* on 8p12, *MDM2* on 12q13, *E2F3*, *CDKAL1*, *SOX4*, *MBOAT1*, *NUP153*, *AOF1*, *FAM8A1*, and *DEK* on 6p22, *CCND1* on 11q13, and *CCNE1* and *BCL2L1* on 20q11 are amplified, while the *CDKN2A* gene located on chromosomal region 9p21.3 is deleted [19, 20, 22]. Furthermore, invasive bladder tumors are characterized by preferential copy number gain of chromosomal regions 7p11.2-q11.22 and 19q13.12-q13.2, and copy number loss of regions 5q14.1-q23.1, 6q14.1-q27, 8p22-p21.3, 11q13.5-q14.1, and 15q11.2-q22.2 [21].

Bladder cancers are classified into two categories, superficial (80%) and muscle-invasive (20%) [3–6]. Non-muscle invasive (superficial) bladder cancer is presented by low-grade tumors with papillary morphology and is characterized with good prognosis (5-year survival rate: 90%). In contrast, muscle invasive (invasive) bladder cancer presents poorly differentiated high-grade tumors demonstrating invasion at the initial presentation to the clinic and has poorer prognosis (5-year survival rate: <50%). Patients with superficial bladder cancer are curatively treated with transurethral resection (TUR), whereas those with muscle invasive bladder cancer undergo radical cystectomy and urinary diversion, or combination chemotherapy [3–6, 23]. Superficial bladder cancer often recurs, and in about 10~30% of patients, it transforms into muscle invasive cancer in the later stage. Currently, there are no prognostic biomarkers for bladder cancer transformation into the invasive phenotype and the probability of recurrence and progression of superficial bladder cancers is calculated based on clinicopathological parameters [24].

*N*-butyl-*N*-(4-hydroxybutyl)nitrosamine (BBN) is a chemical carcinogen which induces bladder cancer in rodent models and *N*-butyl-*N*-(3-carboxybutyl)nitrosamine (BCPN) is a major oxidative metabolite of BBN in the urine, shown to have mutagenic activity and to be involved in bladder carcinogenesis [25–27]. BBN induces superficial papillary tumors in rats and non-papillary invasive tumors in mice. Here, we performed genome-wide analysis of copy number aberrations in BBN-induced bladder cancer in rodent experimental models. Array CGH revealed that several mouse chromosome regions, including the *Cyp2a5* and *Cyp2a22* loci on mouse chromosome 7qA3, were amplified in invasive bladder cancer. Because the mouse *Cyp2a5* gene is the ortholog of the human *CYP2A6* gene, we also evaluated *CYP2A6* copy number and protein expression in resected human bladder cancer specimens.

## Materials and Methods

### Experimental rodent models

Six-week-old Fischer 344 (F344) male rats and C57BL/6 male mice were purchased from Charles River Japan (Atsugi, Japan). Rats and mice were housed in wire and plastic cages, respectively, in an air-conditioned animal room with a 12-h light/dark cycle as described previously [28]. BBN purchased from Tokyo Kasei Kogyo (Tokyo, Japan) was freshly prepared every 2 days as 0.05% solution in tap water. After 1-week acclimatization to the housing environment, mice and rats were provided with a basal diet and water supplemented or not with 0.05% BBN. Rats were treated with BBN for 12 weeks and sacrificed by exsanguination at 28 weeks after cessation of the treatment, and mice were treated with BBN for 4, 8, 12, 20, and 26 weeks and sacrificed by exsanguination immediately after the treatment. Bladder tumors were resected with a razor blade; one half was frozen and stored at -80°C until molecular assays, and the other was fixed in 10% neutral formalin overnight, embedded in paraffin, and cut into 4-μm sections for histological analysis following hematoxylin and eosin staining [29]. Normal bladder tissues of age-matched control rats and mice not treated with BBN were processed in parallel. The study was carried out in strict accordance with the recommendations of the Guide for the Care and Use of Laboratory Animals of the National Institutes of Health. The protocol was approved by the Committee on the Ethics of Animal Experiments of the National Cancer Center Research Institute (Permit Number T05-043). All surgeries were performed under ether anesthesia, and all efforts were made to minimize suffering.

### Human cell lines

The human bladder cancer cell lines RT4, RT112, 5637, T24, J82, HT1197, 253J, and TCCSUP were kindly provided by Dr. Osamu Ogawa (Department of Urology, Kyoto University, Japan) [30–34] in 2007. RT4 and T24 were cultivated in McCoy’s 5A (modified) medium, the others RPMI1640 medium, supplemented with 10% fetal bovine serum.

### Samples of human primary bladder tumors

Primary bladder tumors were resected from patients by total cystectomy (14 samples) or by TUR (104 samples) at the National Cancer Center Hospital of Japan. Resected tumor samples were stored at -80°C for histological or genetic analyses. This study was approved by the Ethics Committee of the National Cancer Center of Japan and was performed in accordance with the Declaration of Helsinki, 1995. All patients gave their written informed consent prior to their inclusion in this study.

### Extraction of genomic DNA

Genomic DNA was extracted from fresh-frozen samples of rodent normal tissues, bladder tumors, and cultured human cell using the Qiagen DNeasy Blood & Tissue Kit (Qiagen, Valencia, CA, USA). Alternatively, genomic DNA was extracted from formalin-fixed, paraffin-embedded human primary bladder tumors after de-paraffinization and tumor dissection using conventional phenol-chloroform extraction method as described previously [35].

### Array CGH

Array CGH analysis was performed using an Agilent platform (Agilent Technologies, Santa Clara, CA, USA) according to the manufacturer’s protocol. Agilent 185K rat genome CGH microarray chips and 244K mouse genome chips were scanned using an Agilent Microarray Scanner, and the data were analyzed using the CGH Analytics Software (Version 3.4) and the DNA Analytics CGH Module (Genomic Workbench Standard Edition 5.0.14), respectively.

### Quantitative PCR

Genomic DNA extracted from mouse tissues and paraffin-embedded human primary bladder tumors was analyzed by qPCR using SYBR Green PCR Master Mix and the 7300 or 7500 real-time PCR systems (Applied Biosystems, Foster City, CA, USA) according to the manufacturer’s instructions. PCR primers for mouse and human genes were designed using the Primer 3 software (www.broadinstitute.org/cgi-bin/primer/primer3_www.cgi) or the Primer Express Version 3.0 software (Applied Biosystems) and evaluated for specificity using in-silico PCR search (S1 Table). Standard curves for mouse and human genes were constructed based on genomic DNA extracted from normal mouse liver and commercially available human (male) genomic DNA (product #G147A 26003101; Promega, Madison, WI, USA), respectively. Copy numbers of mouse and human genes were normalized to those of the mouse L1td1 and human LINE1 sequences, respectively, used as internal controls, and the copy number change was then calculated based on the assumption that normal mouse liver and human genomic DNA is diploid.

The results were validated using genomic DNA extracted from cultured human cells. Gene copy numbers were estimated by qPCR using FAM-labeled probes (Universal Probe Library Set, Human Probe#1–90; Hoffmann-La Roche Ltd, Basel, Switzerland) and TaqMan Gene Expression Master Mix (Applied Biosystems). Primers and probes were designed using the website (https://qpcr.probefinder.com/input.jsp?organism=h_sap), and candidate sequences were examined for homology with other genomic regions and for the presence of known polymorphisms registered in dbSNP (S2 Table). The candidate primers and probes were tested using germline DNA of a healthy woman, and primer-probe combinations were selected based on a standard titration curve with a slope of < -3 and R2 > 0.996. RNase P (Applied Biosystems) was used as an internal control.

qPCR was performed in quadruplicate using an ABI7900HT instrument (Applied Biosystems). Each well contained FAM- and VIC-labeled probes for target genes and RNaseP, respectively, and the related primers. The threshold cycle (Ct) of the FAM probe was adjusted to that of the VIC probe, and ΔCt was calculated. Copy number was estimated according to the ΔCt of human genomic DNA, which was assumed to have two copies of respective genes.

### Immunohistochemistry

Immunohistochemistry was performed using the Histofine Simple Stain MAX PO kit (Nichirei, Tokyo, Japan) according to the manufacturer’s protocol. Rodent CYP2A5 and human CYP2A6 proteins were detected using rabbit polyclonal antibodies against rat CYP2A1 (Nosan, Yokohama, Japan) and human CYP2A6 (Biomol International, Plymouth Meeting, PA, USA), respectively.

Human bladder cancer specimens were classified into those with non-invasive (superficial) papillary lesions (n = 53) and lesions showing invasion into the lamina propria or deeper (n = 240); among the latter, invasive aggregated lesions (n = 108) and invasive scattered lesions (n = 132) were distinguished based on histology. CYP2A6 immunoreactivity was assessed in more than 200 tumor cells in each lesion at magnification ×400, and scored as follows: score 0, no immunoreactivity; score 2, immunoreactivity equal to or stronger than that in the normal liver; and score 1, immunoreactivity between 0 and 2.

### Statistical analysis

Statistical analyses were performed by the Ystat 2002 software (Igaku Tosho Shuppan, Tokyo, Japan) together with Microsoft Excel software. Unpaired Student’s *t*-test was used to evaluate the significance of the difference in relative *CYP2A6* copy numbers in primary bladder tumors and the association between CYP2A6 immunoreactivity and invasive phenotype of human bladder tumors. Correlation between copy number aberrations and CYP2A6 expression in primary bladder tumors was analyzed by parametric test. The difference in CYP2A6 immunoreactivity between incipient and recurrent cases in superficial papillary tumors was evaluated by Mann-Whitney *U*-test, and that between superficial papillary and invasive scattered lesions in the same patients was assessed by paired Student’s *t*-test. Statistical significance was set up at *p* < 0.05.

## Results

### BBN-induced bladder carcinogenesis

**Fig 1:**
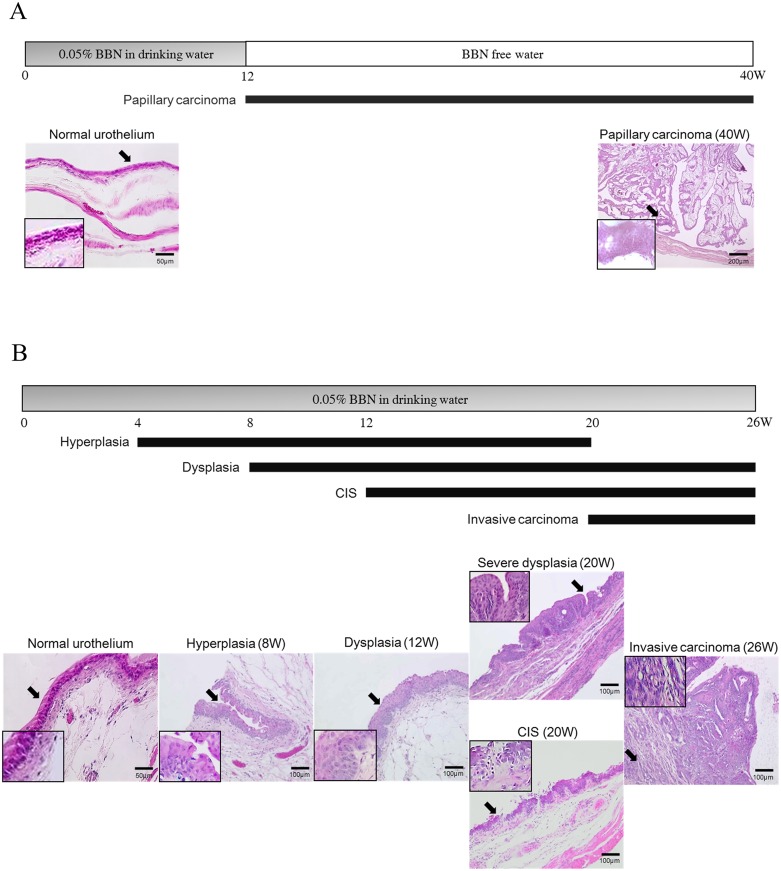
BBN-induced bladder carcinogenesis in rodents. (A) Experimental schedule of bladder cancer induction by BBN in rats (scheme); the time of superficial bladder cancer appearance is indicated. Representative images of hematoxylin/eosin-stained normal urothelium, and papillary carcinoma (week 40) are shown; the inset images show higher magnification. (B) Experimental schedule of bladder cancer induction by BBN in mice (scheme); chronological appearance of hyperplasia, dysplasia, carcinoma *in situ* (CIS), and invasive bladder cancer is indicated. Representative images of hematoxylin/eosin-stained normal urothelium, hyperplasia (week 8), dysplasia (week 12), severe dysplasia (week 20), CIS (week 20), and invasive carcinoma (week 26) are shown; the inset images show higher magnification.

In rats receiving 0.05% BBN for 12 weeks, superficial tumors appeared in the bladder 40 weeks after the start of the treatment (Fig 1A). In mice continuously receiving 0.05% BBN, hyperplasia, dysplasia, carcinoma *in situ* (CIS), and invasive tumors chronologically appeared in the bladder 4, 8, 12, and 20 weeks, respectively, after the start of the treatment (Fig 1B). These results were consistent with previous reports of species-specific phenotypes of BBN-induced bladder cancers in rodents [25–27].

Histological examination revealed that superficial tumors in rats and invasive tumors in mice were of a mixed type containing urothelial carcinomas and squamous cell carcinomas (Fig 1).

### Array CGH analysis of bladder cancer in rodents

Three samples of superficial bladder cancer were obtained from rats at 40 weeks, and two and three samples of invasive bladder cancer were obtained from mice at 20 and 26 weeks, respectively, after the initiation of BBN treatment. Genomic landscapes of rodent bladder tumors were compared to those of normal tissues from respective species using array CGH, and the ratios of gene copy numbers in tumors relative to normal tissues for microarray probes were converted to log_2_ values. Chromosomal regions showing significant copy number aberrations in bladder tumors were screened based on the following strict criteria: (i) log_2_ ratio more than +1 (copy number > 4) or less than -1 (copy number < 1) in more than 50% of invasive or superficial tumors; (ii) log_2_ ratio more than +1 or less than -1 for at least two adjacent microarray probes.

**Fig 2:**
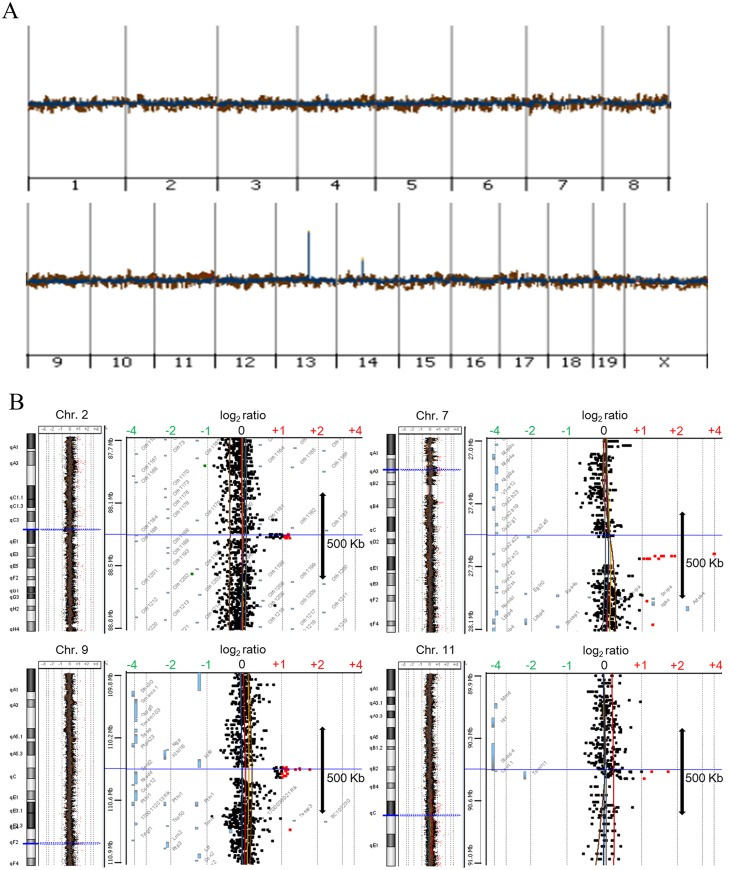
Array CGH analysis of invasive bladder tumors in BBN-treated mice. (A) Whole-genome array CGH profile. (B) Detailed array CGH profile of mouse chromosomal regions 2D-E1, 7qA3, 9F2, and 11C-D. Tumor to normal copy number ratios for microarray probes expressed as log_2_ values are shown by dots. Red dots indicate significant copy number gain (log_2_ ratio > +1).

Based on these screening criteria, no significant copy number aberrations were detected in BBN-induced superficial bladder tumors in rats (data not shown). However, copy number gain or gene amplification of chromosomal regions 2D-E1, 7qA3, 9F2, and 11C-D were detected in BBN-induced invasive bladder tumors in mice, although no significant copy number loss was observed (Fig 2).

### Validation of array CGH data by qPCR

To validate array CGH data on the amplified regions, copy number changes of the *Olfr1184* and *Rapsn* genes on mouse chromosomal region 2D-E1, the *Cyp2g1*, *Cyp2a5*, *Cyp2a22*, *Cyp2a12*, and *Rab4b* genes on 7qA3, the *Klhl18*, *Kif9*, and *Nradd* genes on 9F2, and *Stxbp4* and *Cox11* genes on 11C-D were investigated by qPCR in 2 and 4 samples of invasive bladder tumors obtained at 20 and 26 weeks, respectively, after the initiation of BBN treatment (S3 Table). Gene amplification by qPCR was defined as copy number gain > +1 (3 or more).

**Fig 3:**
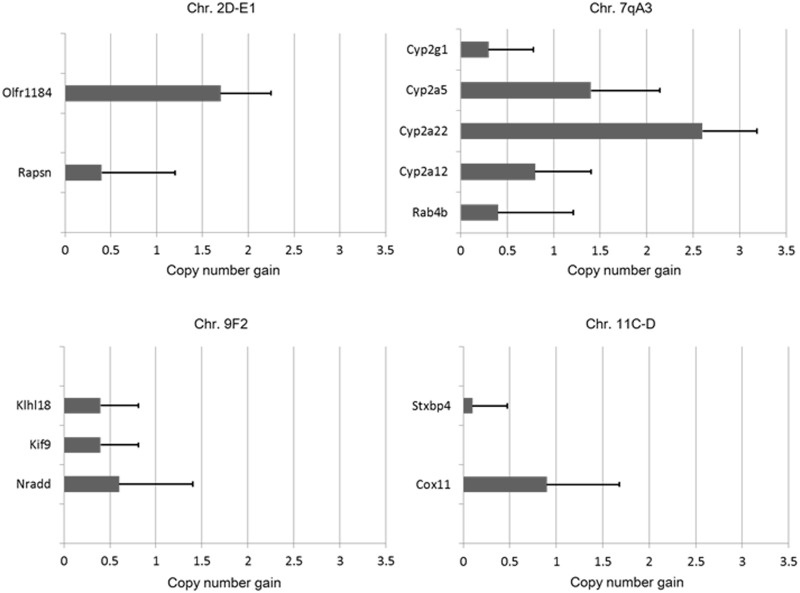
Validation of the array CGH results by qPCR. Copy number changes of representative genes, including the amplicons on mouse chromosomal regions 2D-E1, 7qA3, 9F2, and 11C-D, are shown.

The results confirmed that the *Olfr1184* gene on 2D-E1, and *Cyp2a5* and *Cyp2a22* genes on 7qA3 were amplified in mouse invasive bladder tumors (Fig 3).

### Syntenic comparison of amplicons in mouse and human bladder tumors

Mouse chromosomal regions 2D-E1 and 7qA3 are syntenic to human chromosomal regions 11p11.2-q12.1 and 19q13.2, respectively. Amplification of the 11p11.2-q12.1 region was not detected in a previous array CGH study on human bladder cancer [19–22]. However, another study has reported amplification of the *DPF-PPP1R14A-SPINT2-KCNK6* and *ACTN4* loci on chromosome 19q13 in human bladder tumors [20] and of the chromosomal region19q13.12-q13.2 in human urothelial carcinomas, especially in invasive types [21, 22].

The human *CYP2A6* gene on chromosomal region 19q13.2 is the ortholog of mouse *Cyp2a5*. The human ortholog gene of *Cyp2a22*, which was the major amplified gene on mouse 7qA3 (Figs 2 and 3), is not confirmed at present. However, *CYP2A6* was detected as a human gene homologous to *Cyp2a22* by searching Ortho DB (the Hierarchical Catalog of Orthologs ***v8***) and was identified as one of the closest *Cyp2a22*-related human genes according to BlastP search of AceView. Therefore, we considered a possibility that mouse 7qA3 was homologous to human 19q13.2. Consistent with a previous study on tumorigenic mechanisms, which investigated *Cyp2a5* expression in mouse lung adenoma [36], we detected CYP2A immunoreactivity in surface urothelial cells of the non-cancerous portion of the rodent bladder (regarded as non-specific). We also found that CYP2A expression was slightly higher in dysplasia and significantly higher in invasive lesions compared to basal cells in the normal bladder lamina propria (S1 Fig). Human *CYP2A6* clustered with *CYP2A7*, *CYP2B6*, *CYP2A13*, and *CYP2F1* is distant from the *DPF-PPP1R14A-SPINT2-KCNK6* and *ACTN4* loci within gene-rich chromosomal region 19q13.

The *CYP2A6* gene polymorphism has been found to be associated with the development of lung cancer [37], and *CYP2A6* has emerged as a novel candidate oncogene [38]; however, *CYP2A6* amplification has not been investigated. Therefore, we next examined *CYP2A6* copy number and protein expression in human bladder cancer.

### Amplification of the *CYP2A6* gene in human bladder cancer in vitro and in vivo

**Fig 4:**
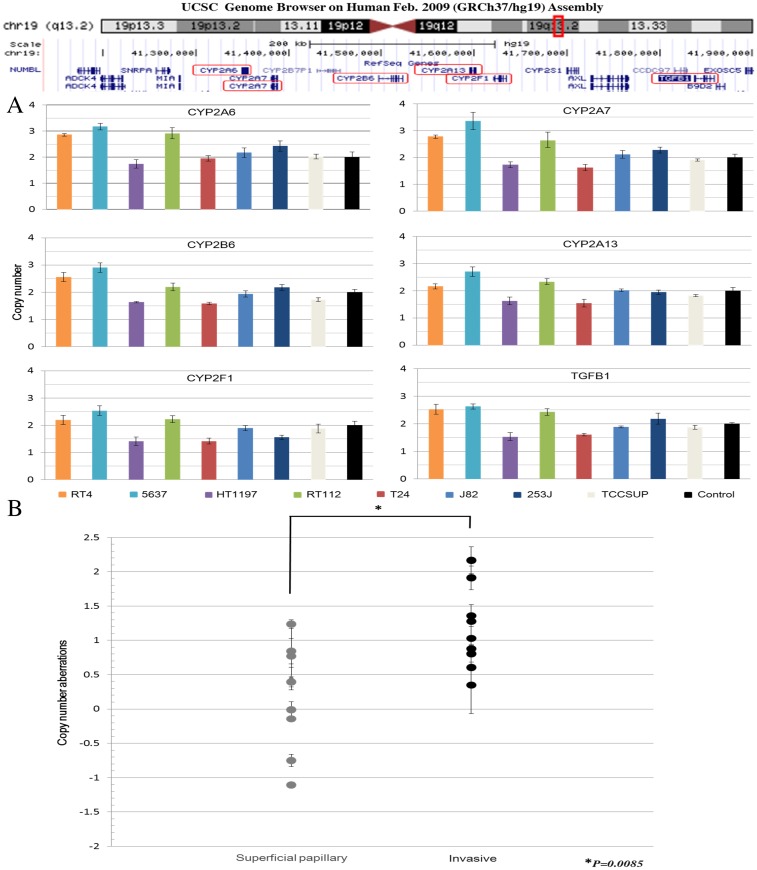
Copy number aberrations of the *CYP2A6* and neighboring genes in human bladder cancer. (A) Copy numbers of *CYP2A6*, *CYP2A7*, *CYP2B6*, *CYP2A13*, *CYP2F1*, and *TGFB1* on human chromosomal region 19q13.2 in eight bladder cancer cell lines. (B) *CYP2A6* amplification in primary bladder tumors. *CYP2A6* copy number aberrations in each case of superficial (n = 9) and invasive (n = 9) bladder cancers are shown.

We analyzed copy number aberrations of the *CYP2A6* and neighboring genes in human bladder cancer cell lines RT4, RT112, 5637, T24, J82, HT1197, 253J, and TCCSUP. The amplification of *CYP2A6* and *CYP2A7* (copy number 3 or more) could be merely detected in one bladder cancer cell line (Fig 4A).

The amplification of the *CYP2A6* gene in primary bladder cancers was also tested by qPCR which confirmed that *CYP2A6* was amplified in one papillary tumor and five invasive tumors (copy number gain > +1), and the difference was statistically significant (p = 0.0085; Fig 4B). These results indicate that *CYP2A6* copy number is significantly higher in invasive compared to superficial bladder tumors.

### Overexpression of the CYP2A6 protein in human bladder cancer

**Fig 5:**
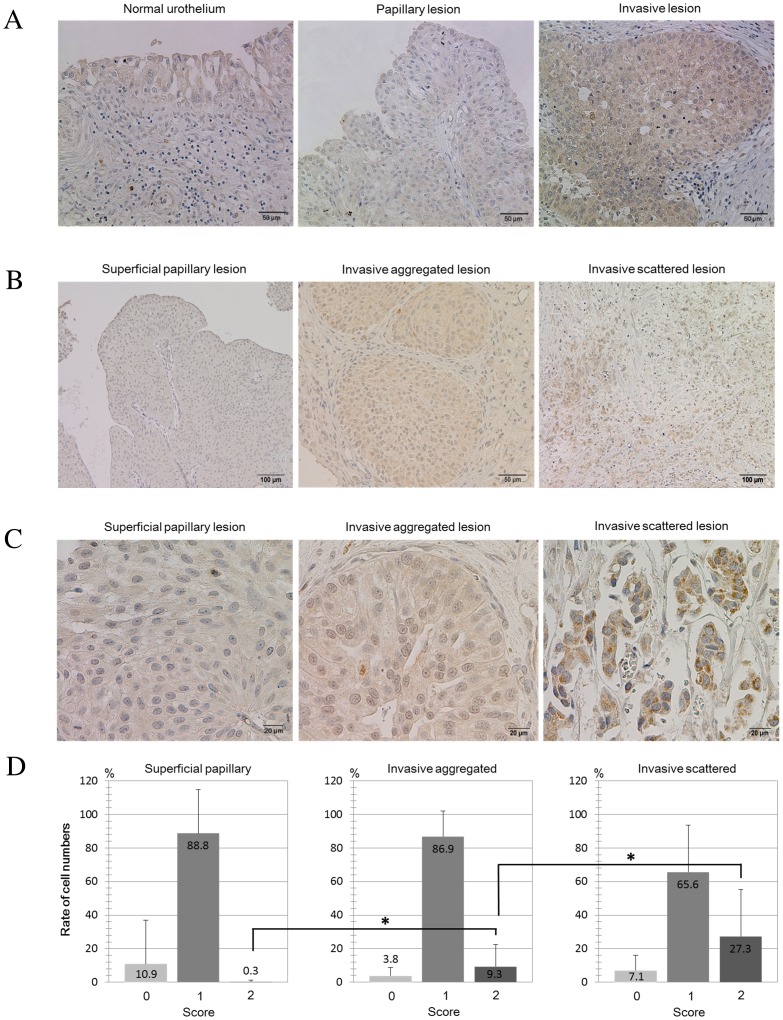
Immunohistochemical analysis of CYP2A6 protein expression in human bladder cancer. (A) CYP2A6 immunoreactivity in a representative primary bladder tumor containing superficial and invasive lesions. (B and C) CYP2A6 distribution in indicated types of lesions. Images are obtained at low (B) and high (C) magnification. (D) Quantification of CYP2A6 immunoreactivity in primary bladder cancers. Primary bladder tumors (n = 104) were classified into those with superficial papillary lesions (n = 53), invasive aggregated lesions (n = 108), and invasive scattered lesions (n = 132). *p < 0.01 (invasive aggregated versus superficial papillary lesions and invasive scattered versus invasive aggregated lesions; Student’s *t*-test).

Immunohistochemical analysis was initially performed on 14 primary bladder tumors resected by total cystectomy. CYP2A6 immunoreactivity was detected in surface urothelial cells of the non-cancerous portion of the bladder, and was found upregulated in invasive lesions of primary tumors (Fig 5A).

To investigate the association between CYP2A6 expression and invasive phenotype, immunohistochemical analysis was performed in 104 primary bladder tumors resected by TUR (S4 Table). Because some samples showed intra-tumor heterogeneity and presented lesions with distinct phenotypes, bladder cancer tumors were classified into those containing superficial papillary lesions, invasive aggregated lesions, and invasive scattered lesions (Fig 5B and 5C). Among superficial papillary, invasive aggregated, and invasive scattered lesions, 0.3%, 9.3%, and 27.3% cells, respectively, demonstrated high (score 2) CYP2A6 expression (Fig 5D), and the difference among the lesions was significant (p < 0.01). These data clearly indicate that the upregulation of CYP2A6 expression was associated with the histologically invasive phenotype of human bladder tumors. Analysis of the correlation between *CYP2A6* copy number aberrations and its expression in superficial and invasive cancers indicated that only invasive cancers exhibited positive correlation between copy number gain and increased expression (superficial; *r* = -0.47, *P* = 0.098, invasive; *r* = 0.59, *P* = 0.048 by parametric test; S2 Fig).

To analyze the role of CYP2A6 in tumor progression, CYP2A6 expression was examined in incipient and recurrent TUR-treated cases in superficial papillary tumors, and in superficial papillary and invasive scattered lesions present in the same patient (S3 Fig, S4 and S5 Tables). In superficial papillary tumors, the difference in CYP2A6 expression estimated based on immunohistochemical score and cell number ratio was not statistically significant, suggesting that CYP2A6 expression was not increased in superficial papillary tumors even after relapse. Among 40 cases of invasive scattered lesions treated by TUR, 8 also had superficial papillary lesions in the same specimen; these cases demonstrated a significant difference in CYP2A6 expression (the ratio of cells scored 2 by CYP2A6 immunoreactivity) between superficial papillary and invasive scattered lesions. This result corroborates our previous findings, further supporting the conclusion that CYP2A6 overexpression is associated with the transformation of superficial papillary tumors to invasive phenotype.

## Discussion

This study investigated genomic landscapes in chemically induced bladder cancers of rodents. BBN treatment induced bladder cancers of superficial and invasive types in rats and mice, respectively, as described previously [25–27]. According to strict in-house criteria for array CGH analysis, no significant copy number alteration was detected in BBN-induced superficial bladder tumors in rats; however, copy number gains on chromosomal regions 2D-E1, 7qA3, 9F2, and 11C-D were detected in BBN-treated invasive bladder cancers in mice. When array CGH data were validated by qPCR analysis of copy numbers for representative genes in each amplicon, *Olfr1184* on chromosome 2D and *Cyp2a22* on chromosome 7qA3 demonstrated amplification in mouse invasive bladder cancers. *Cyp2a5* copy number was not more than +1 by CGH analysis; however, the amplification was detected by qPCR. The discrepancy may be partly due to the fact that the region amplified by qPCR using our *Cyp2a5* primer (27,624,139–27,624,316) was closer to the *Cyp2a22* locus by about 3,500 bp compared to that targeted by the CGH probe (27,620,744–27,620,803).

We then performed syntenic comparison of human and mouse bladder tumors. The *CYP2A6* locus on human chromosomal region 19q13.2 is syntenic to the *Cyp2a5* locus on mouse chromosomal region 7qA3. Blaveri et al. have reported that the *SPINT2* and *ACTN4* loci on human 19q13 are amplified in bladder cancers [20], while Lindgren et al. have found copy number gain of chromosomal region 19q13.12-q13.2 [22], which has been shown to be associated with aggressive phenotype in invasive bladder cancer [21]. Because the *CYP2A6* locus in the 19q13 region is separated from the *SPINT2* and *ACTN4* loci by many genes, *CYP2A6* amplification in human cancer has not been shown. In addition, it is also possible that the CYP2A5 protein was weakly expressed in BBN-induced mouse dysplasia and had stronger expression in mouse invasive cancers by immunohistochemistry. Therefore, we investigated *CYP2A6* gene amplification and protein expression in human bladder cancer.

The amplification of the *CYP2A6* locus was not clearly detected in human bladder cancer cell lines, which might be due to cell line origin from fully metastasized cancer. However, the *CYP2A6* gene was amplified in six out of 18 cases of resected primary bladder tumors, suggesting an association with the early stage of cancer invasion. A search of available public databases, such as GEO and Oncomine, did not produce any results on the correlation of CYP2A6 expression levels with bladder cancer disease stage. According to cBioPortal, *CYP2A6* amplification frequency was 4.7% in 128 cases (TCGA, Nature 2014) [39], 2.7% in 408 cases (TCGA, Provisional), and 1% in 97 cases (MSKCC, J Clin Oncol 2013) [40]. We consider that the difference between our data on TUR samples and other databases may be due to the heterogeneity of bladder cancers. Thus, in urothelial cancers, heterogeneity of cellular atypia is frequently observed by histology: either a small area showing a higher grade cellular atypia develops within low-grade tumors, or cancer cells gain higher grade cellular atypia before they start to disrupt the basal membrane and invade into subepithelial tissues [21]. We extracted genomic DNA from superficial papillary, invasive aggregated, and invasive scattered lesions of TUR specimens at pinpoint under the microscope. It is feasible that copy number heterogeneity corresponds to histological heterogeneity during the multistep malignant progression of urothelial cancers [21]; therefore, the ratio of CYP2A6 amplification observed in our study may be higher than that reported in public datasets. Stratified analysis further revealed that *CYP2A6* amplification preferentially occurred in invasive bladder cancer. These results indicate that the amplification of the *CYP2A6* gene could be involved in the development of invasive bladder cancer phenotype. This notion was confirmed by significant upregulation of the CYP2A6 protein in invasive bladder tumors, especially in scattered lesions. In addition, only invasive cancers exhibited positive correlation between *CYP2A6* copy number gains and increased expression. Moreover, CYP2A6 immunoreactivity was significantly different between superficial papillary and invasive scattered lesions in the same cases, although such difference was not observed between incipient and recurrent cases of superficial papillary tumors. Together, these data suggest that *CYP2A6* gene amplification and overexpression are associated with malignancy in human bladder cancer. To the best of our knowledge, there are no reports about immunohistochemistry analysis of CYP2A6 in human bladder cancers. Therefore, our immunohistochemistry experiments were based on the studies that performed CYP2A6 immunohistochemistry in other human cancers [41, 42]. Antibodies recognizing CYP proteins are usually raised against their C-terminal domains [43]. However, CYP2A6 has high sequence similarity with CYP2B6; in particular, their C-termini are identical in four out of five residues [43]. Consequently, the anti-CYP2A6 antibody can also recognize CYP2B6, suggesting that the involvement of CYP2B6 cannot be ruled out.

CYP2A6 belongs to the CYP family of cytochrome P450 enzymes implicated in the metabolism of drugs and environmental carcinogens, especially nicotine in cigarette smoke [44–47]. Given that cigarette smoking is an environmental risk factor for bladder cancer [10], germ-line polymorphism of the *CYP2A6* gene might be associated with an increased risk of this type of malignancy related with cigarette smoke. A small-scale case-control study has revealed the association of CYP2A6*1/*4 or *4/ *4 genotypes with decreased susceptibility to bladder cancer [48]; however, the relationship between *CYP2A6* SNPs and bladder cancer risk has not been demonstrated by GWASs [13–17]. Recently, Matsuda et al. have reported that CYP1A2, another member of the CYP family of enzymes thought to metabolize tobacco-derived carcinogens, is associated with bladder cancer risk in Japanese population [18]. However, that study did not discriminate between non-invasive and invasive bladder cancers; therefore, CYP2A6 contribution to the risk of developing invasive bladder cancer phenotype could not be clearly determined. Nevertheless, previous findings indicate significant involvement of CYP family members in bladder cancer progression, which is consistent with our results.

Human cancers develop in a step-wise manner depending on the accumulation of multiple epigenetic changes and genetic alterations [1, 2]. Gene amplification is one of the mechanisms underlying the activation of proto-oncogenes such as *ERBB2*, *CCND1*, *CCNE1*, *MDM2*, and *E2F3*, which have been suggested as cancer driver genes in bladder cancers, based on their aberrant gene amplification and protein overexpression [5,19–22]. In bladder cancer, the amplification and overexpression of the *CYP2A6* gene was associated with increased malignancy, namely invasiveness, both in rodents and in humans. Although previous studies have demonstrated the upregulation of CYP2A6 protein and mRNA in human lung cancer and colorectal cancer [41, 42], this is the first report on *CYP2A6* gene amplification and overexpression in bladder cancer.

However, our study has some limitations. First, our data do not demonstrate that gene amplification is directly associated with the overexpression of Cyp2a5/CYP2A6. To explore the correlation between *CYP2A6* amplification and overexpression in human bladder cancer, we searched the Catalogue of Somatic Mutations in Cancer (COSMIC). The results indicate that 4 out of 113 of bladder cancer samples (3.54%) had copy number gain, and 2 of them (50%) demonstrated CYP2A6 overexpression, suggesting that CYP2A6 protein levels may be regulated by other mechanisms at the transcriptional, translational, or epigenetic levels. However, it should be mentioned that we did not find bladder tumors with superficial phenotype investigated for copy number aberrations (CNAs) and protein expression of Cyp2a5/CYP2A6, but only those with invasive phenotype, both in rodents and humans. Furthermore, a recent report on proteogenomics of human colon and rectal cancers indicated that focal CNAs have the strongest *cis*-effects on both mRNA and protein levels, suggesting that selection for high protein expression may promote CNA in the regions of focal amplification [49]. Therefore, we consider that the association of invasive bladder cancer phenotype with the amplification and potential overexpression of *Cyp2a5/CYP2A6* could be highly plausible.

Another limitation is the specificity of the anti-CYP2A6 antibody. Unfortunately, currently available commercial antibodies against CYP2A6 have low specificity and cannot be reliably used for investigating CYP2A6 protein levels by immunohistochemistry as evidenced by our search of public databases. Therefore, our results on CYP2A6 protein expression in bladder cancer should be further validated using an antibody recognizing CYP2A6 with high specificity and sensitivity.

Although our data indicate possible involvement of CYP2A6 in bladder cancer progression with invasive phenotype, it should be validated in future experimental and clinical studies.

Bladder tumors are pathologically heterogeneous and consist of superficial lesions with papillary morphology and invasive lesions with malignant phenotype [3–6]. Recurrence of cancer invasion after TUR-based curative resection of superficial bladder tumors is a serious clinical problem, and predictive biomarkers associated with invasion recurrence are necessary for appropriate clinical care of bladder cancer patients. Although the analyzed number of tumors was limited, our results suggest that CYP2A6 is a candidate prognostic biomarker that can be utilized for therapeutic stratification of patients with bladder cancers. Large-scale clinical trials are necessary to validate CYP2A6 potential as a useful biomarker for early detection of invasive phenotype of bladder cancer.

## Supporting Information

S1 Fig Immunohistochemical examination of CYP2A expression in the rodent bladder normal urothelium, dysplasia, and superficial and invasive lesions induced by BBN. (TIF)

S2 Fig Correlation between copy number aberrations and CYP2A6 expression in superficial and invasive bladder cancers. (TIF)

S3 Fig CYP2A6 immunoreactivity in incipient and recurrent cases of superficial papillary tumors, and in superficial papillary and invasive scattered lesions in the same cases. (A) CYP2A6 immunoreactivity in incipient (n = 18) and recurrent (n = 27) cases of superficial papillary tumors. The mean value based on the immunohistochemical score and cell number ratio in each TUR sample is shown. *p = 0.196 (incipient versus recurrent cases; Mann-Whitney *U*-test, 95th, 75th, 50th, 25th, and 10th percentile). (B) CYP2A6 immunoreactivity in 8 paired samples of superficial papillary and invasive scattered lesions derived from the same patient. The ratio of cells with score 2 in the same case is shown. Black and white circles represent a data plot and a mean plot, respectively. **p = 0.0162 (superficial papillary versus invasive scattered lesions; paired *t*-test). (C) CYP2A6 immunoreactivity in a representative case demonstrating superficial papillary and invasive scattered lesions.
(TIF)

S1 Table Primer sequences for qPCR analysis of mouse and human primary bladder tumors. (DOCX)

S2 Table Primer sequences for qPCR analysis of human cell lines. (DOCX)

S3 Table Copy number changes investigated by qPCR. (DOCX)

S4 Table Immunohistochemical analysis of 104 primary bladder tumors resected by TUR. (DOCX)

S5 Table CYP2A6 expression in superficial papillary lesions found among TUR samples of invasive scattered lesions present in the same patient. (DOCX)

## References

[1] Sugimura T. Multistep carcinogenesis: a 1992 perspective. Science 1992;258: 603–607. 1411570 10.1126/science.1411570

[2] Katoh M. Network of WNT and other regulatory signaling cascades in pluripotent stem cells and cancer stem cells. Curr Pharm Biotechnol. 2011;12: 160–170. 21044011 10.2174/138920111794295710

[3] Blaveri E, Simko JP, Korkola JE, Brewer JL, Baehner F, Mehta K, et al Bladder cancer outcome and subtype classification by gene expression. Clin Cancer Res. 2005;11: 4044–4055. 10.1158/1078-0432.CCR-04-2409 15930339

[4] Sugano K, Kakizoe T. Genetic alterations in bladder cancer and their clinical applications in molecular tumor staging. Nat Clin Pract Urol. 2006; 3: 642–652. 10.1038/ncpuro0649 17149381

[5] Knowles MA. Molecular subtypes of bladder cancer: Jekyll and Hyde or chalk and cheese? Carcinogenesis 2006;27: 361–373. 10.1093/carcin/bgi310 16352616

[6] Babjuk M, Burger M, Zigeuner R, Shariat SF, van Rhijn BW, Comérat E, et al EAU guidelines on non-muscle-invasive urothelial carcinoma of the bladder: update 2013. Eur Urol. 2013;64: 639–653. 10.1016/j.eururo.2013.06.003 23827737

[7] Lichtenstein P, Holm NV, Verkasalo PK, Iliadou A, Kaprio J, Koskenvuo M, et al Environmental and heritable factors in the causation of cancer—analyses of cohorts of twins from Sweden, Denmark, and Finland. N Engl J Med. 2000;343: 78–85. 10.1056/NEJM200007133430201 10891514

[8] Chu H, Wang M, Zhang Z. Bladder cancer epidemiology and genetic susceptibility. J Biomed Res. 2013;27: 170–178. 10.7555/JBR.27.20130026 23720672 PMC3664723

[9] Sampson JN, Wheeler WA, Yeager M, Panagiootou O, Wang Z, Berndt SI, et al Analysis of heritability and shared heritability based on genome-wide association studies for 13 cancer types. J Natl Cancer Inst. 2015;107:10.1093/jnci/djv279 PMC4806328 26464424

[10] Zeegers MP, Tan FE, Dorant E, van den Brandt PA. The impact of characteristics of cigarette smoking on urinary tract cancer risk: a meta-analysis of epidemiologic studies. Cancer 2000;89: 630–639. 10931463 10.1002/1097-0142(20000801)89:3<630::aid-cncr19>3.3.co;2-h

[11] Zeegers MP, Swaen GM, Kant I, Goldbohm RA, van den Brandt PA. Occupational risk factors for male bladder cancer: results from a population based case cohort study in the Netherlands. Occup Environ Med. 2001;58: 590–596. 10.1136/oem.58.9.590 11511746 PMC1740187

[12] Bell DA, Taylor JA, Paulson DF, Robertson CN, Mohler JL, Lucier GW. Genetic risk and carcinogen exposure: a common inherited defect of the carcinogen-metabolism gene *glutathione S-transferase M1 (GSTM1)* that increases susceptibility to bladder cancer. J Natl Cancer Inst. 1993;85: 1159–1164. 8320745 10.1093/jnci/85.14.1159

[13] Kiemeney LA, Thorlacius S, Sulem P, Geller F, Aben KK, Stacey SN, et al Sequence variant on 8q24 confers susceptibility to urinary bladder cancer. Nat Genet. 2008;40: 1307–1312. 10.1038/ng.229 18794855 PMC4539560

[14] Wu X, Ye Y, Kiemeney LA, Sulem P, Rafnar T, Seminara D, et al Genetic variation in the prostate stem cell antigen gene *PSCA* confers susceptibility to urinary bladder cancer. Nat Genet. 2009;41: 991–995. 10.1038/ng.421 19648920 PMC3313685

[15] Kiemeney LA, Sulem P, Besenbacher S, Vermeulen SH, Sigurdsson A, Thorleifsson G, et al A sequence variant at 4p16.3 confers susceptibility to urinary bladder cancer. Nat Genet. 2010;42: 415–419. 10.1038/ng.558 20348956 PMC2923020

[16] Rothman N, Garcia-Closas M, Chatterjee N, Malats N, Wu X, Figueroa JD, et al A multi-stage genome-wide association study of bladder cancer identifies multiple susceptibility loci. Nat Genet. 2010;42: 978–984. 10.1038/ng.687 20972438 PMC3049891

[17] Figueroa JD, Ye Y, Siddiq A, Garcia-Closas M, Chatterjee N, Prokunina-Olsson L, et al Genome-wide association study identifies multiple loci associated with bladder cancer risk. Hum Mol Genet. 2014;23: 1387–1398. 10.1093/hmg/ddt519 24163127 PMC3919005

[18] Matuda K, Takahashi A, Middlebrooks CD, Obara W, Nasu Y, Inoue K, et al Genome-wide association study identified SNP on 15q24 associated with bladder cancer risk in Japanese population. Hum Mol Genet. 2015;24: 1177–1184. 10.1093/hmg/ddu512 25281661 PMC4834880

[19] Veltman JA, Fridlyand J, Pejavar S, Olshen AB, Korkola JE, DeVries S, et al Array-based comparative genomic hybridization for genome-wide screening of DNA copy number in bladder tumors. Cancer Res. 2003;63: 2872–2880. 12782593

[20] Blaveri E, Brewer JL, Roydasgupta R, Fridlyand J, DeVries S, Koppie T, et al Bladder cancer stage and outcome by array-based comparative genomic hybridization. Clin Cancer Res. 2005;11: 7012–7022. 10.1158/1078-0432.CCR-05-0177 16203795

[21] Nishiyama N, Arai E, Nagashio R, Fujimoto H, Hosoda F, Shibata T, et al Copy number alterations in urothelial carcinomas: their clinicopathological significance and correlation with DNA methylation alterations. Carcinogenesis 2011;32: 462–469. 10.1093/carcin/bgq274 21177765 PMC3066412

[22] Lindgren D, Sjödahl G, Lauss M, Staaf J, Chebil G, Lövgren K, et al Integrated genomic and gene expression profiling identifies two major genomic circuits in urothelial carcinoma. PLoS One 2012;7: e38863 10.1371/journal.pone.0038863 22685613 PMC3369837

[23] Witjes JA, Comérat E, Cowan NC, De Santis M, Gakis G, Lebret T, et al EAU guidelines on muscle-invasive and metastatic bladder cancer: summary of the 2013 guidelines. Eur Urol. 2014;65: 778–792. 10.1016/j.eururo.2013.11.046 24373477

[24] Vedder MM, Márquez M, de Bekker-Grob EW, Calle ML, Dyrskjøt L, Kogevinas M, et al Risk prediction scores for recurrence and progression of non-muscle invasive bladder cancer: an international validation in primary tumours. PLoS One. 2014;9: e96849 10.1371/journal.pone.0096849 24905984 PMC4048166

[25] Hashimoto Y, Suzuki E, Okada M. Induction of urinary bladder tumors in ACI-N rats by butyl(3-carboxypropyl)nitrosoamine, a major urinary metabolite of butyl(4-hydroxybutyl)nitrosoamine. Gann 1972;63: 637–638. 4645279

[26] Hashimoto Y, Suzuki K, Okada M. Induction of urinary bladder tumors by intravesicular instillation of butyl(4-hydroxybutyl)nitrosoamine and its principal urinary metabolite, butyl(3-carboxypropyl)nitrosoamine in rats. Gann 1974;65: 69–73. 4825391

[27] Okada M, Ishidate M. Metabolic fate of N-n-butyl-N-(4-hydroxybutyl)-nitrosamine and its analogues: selective induction of urinary bladder tumours in the rat. Xenobiotica 1977;7: 11–24. 191997 10.3109/00498257709036241

[28] Ochiai M, Ushigome M, Fujiwara K, Ubagai T, Kawamori T, Sugimura T, et al Characterization of dysplastic aberrant crypt foci in the rat colon induced by 2-amino-1-methyl-6-phenylimidazo[4,5-b]pyridine. Am J Pathol. 2003;163: 1607–1614. 10.1016/S0002-9440(10)63517-1 14507667 PMC1868301

[29] Fujiwara K, Ochiai M, Ohta T, Ohki M, Aburatani H, Nagao M, et al Global gene expression analysis of rat colon cancers induced by a food-borne carcinogen, 2-amino-1-methyl-6-phenylimidazo[4,5-b]pyridine. Carcinogenesis 2004;25: 1495–1505. 10.1093/carcin/bgh155 15059925

[30] Mizutani Y, Yoshida O, Miki T, Bonavida B. Synergistic cytotoxicity and apoptosis by Apo-2 ligand and adriamycin against bladder cancer cells. Clin Cancer Res. 1999;5: 2605–2612. 10499639

[31] Mizutani Y, Kamoi K, Ukimura O, Kawauchi A, Miki T. Synergistic cytotoxicity and apoptosis of JTE-522, a selective cyclooxygenase-2 inhibitor, and 5-fluorouracil against bladder cancer. J urol. 2002;168: 2650–2654. 10.1097/01.ju.0000030150.25914.c6 12442003

[32] Matsui Y, Ueda S, Watanabe J, Kuwabara I, Ogawa O, Nishiyama H. Sensitizing effect of galectin-7 in urothelial cancer to cisplatin through the accumulation of intracellular reactive oxygen species. Cancer Res. 2007;67: 1212–1220. 10.1158/0008-5472.CAN-06-3283 17283157

[33] Kawanishi H, Matsui Y, Ito M, Watanabe J, Takahashi Y, Nishizawa K, et al Secreted CXCL1 is a potential mediator and marker of the tumor invasion of bladder cancer. Clin Cancer Res. 2008;14: 2579–2587. 10.1158/1078-0432.CCR-07-1922 18451219

[34] Saito R, Shirakawa R, Nishiyama H, Kobayashi T, Kawato M, Kanno T, et al Downregulation of Ral GTPase-activating protein promotes tumor invasion and metastasis of bladder cancer. Oncogene. 2013;32: 894–902. 10.1038/onc.2012.101 22450745

[35] Nakagama H, Kaneko S, Shima H, Inamori H, Fukuda H, Kominami R, et al Induction of minisatellite mutation in NIH 3T3 cells by treatment with the tumor promoter okadaic acid. Proc Natl Acad Sci USA. 1997;94: 10813–10816. 9380716 10.1073/pnas.94.20.10813 PMC23496

[36] Miyazaki M, Yamazaki H, Takeuchi H, Saoo K, Yokohira M, Masumura K, et al Mechanisms of chemopreventive effects of 8-methoxypsoralen against 4-(methylnitrosamino)-1-(3-pyridyl)-1-butanone-induced mouse lung adenomas. Carcinogenesis. 2005;26: 1947–1955. 10.1093/carcin/bgi156 15958517

[37] London SJ, Idle JR, Daly AK, Coetzee GA. Genetic variation of CYP2A6, smoking, and risk of cancer. Lancet. 1999;353: 898–899. 10.1016/S0140-6736(98)04984-8 10093988

[38] Nakada T, Kiyotani K, Iwano S, Uno T, Yokohira M, Yamakawa K, et al Lung tumorigenesis promoted by anti-apoptotic effects of cotinine, a nicotine metabolite through activation of PI3K/Akt pathway. J Toxicol Sci. 2012;37: 555–563. 22687995 10.2131/jts.37.555

[39] Weinstein JN, Akbani R, Broom BM, Wang W, Verhaak RG, McConkey D, et al Comprehensive molecular characterization of urothelial bladder carcinoma. Nature. 2014;507: 315–322. 10.1038/nature12965 24476821 PMC3962515

[40] Iyer G, Al-Ahmadie H, Schultz N, Hanrahan AJ, Ostrovnaya I, Balar AV, et al Prevalence and co-occurrence of actionable genomic alterations in high-grade bladder cancer. J Clin Oncol. 2013;31: 3133–3144. 10.1200/JCO.2012.46.5740 23897969 PMC3753703

[41] Matsuda Y, Yamakawa K, Saoo K, Hosokawa K, Yokohira M, Kuno T, et al CYP2A6 overexpression in human lung cancers correlates with a high malignant status. Oncol Rep. 2007;18: 53–57. 17549345

[42] Matsuda Y, Saoo K, Yamakawa K, Yokohira M, Suzuki S, Kuno T, et al Overexpression of CYP2A6 in human colorectal tumors. Cancer Sci. 2007;98: 1582–1585. 10.1111/j.1349-7006.2007.00572.x 17683511 PMC11159343

[43] Edwards RJ, Boobis AR, Davies DS. A strategy for investigating the CYP superfamily using targeted antibodies is a paradigm for functional studies. Drug Metab Dispos. 2003;31: 1476–1480. 10.1124/dmd.31.12.1476 14625344

[44] Fernandez-Salguero P, Hoffman SM, Cholerton S, Mohrenweiser H, Raunio H, Rautio A, et al A genetic polymorphism in coumarin 7-hydroxylation: sequence of the human *CYP2A* genes and identification of variant CYP2A6 alleles. Am J Hum Genet. 1996;57: 651–660. PMC1801261 7668294

[45] Nakajima M, Yamamoto T, Nunoya K, Yokoi T, Nagashima K, Inoue K, et al Role of human cytochrome P4502A6 in C-oxidation of nicotine. Drug Metab Dispos. 1996;24: 1212–1217. 8937855

[46] Messina ES, Tyndale RF, Sellers EM. A major role for CYP2A6 in nicotine C-oxidation by human liver microsomes. J Pharmacol Exp Ther. 1997;282: 1608–1614. 9316878

[47] Agundez JA. Cytochrome P450 gene polymorphism and cancer. Curr Drug Metab. 2004;5: 211–224. 15180491 10.2174/1389200043335621

[48] Song DK, Xing DL, Zhang LR, Li ZX, Liu J, Qiao BP. Association of *NAT2*, *GSTM1*, *GSTT1*, *CYP2A6*, and *CYP2A13* gene polymorphisms with susceptibility and clinicopathologic characteristics of bladder cancer in central China. Cancer Detect Prev. 2009;32: 416–423. 10.1016/j.cdp.2009.02.003 19303722

[49] Zhang B, Wang J, Wang X, Zhu J, Liu Q, Shi Z, et al Proteogenomic characterization of human colon and rectal cancer. Nature. 2014;513: 382–387. 10.1038/nature13438 25043054 PMC4249766

