# Supplementary figures and images for: Genomic Landscape of Experimental Bladder Cancer in Rodents and Its Application to Human Bladder Cancer: Gene Amplification and Potential Overexpression of Cyp2a5/CYP2A6 Are Associated with the Invasive Phenotype
Source: PLoS One. 2016 Nov 30;11(11):e0167374. doi: 10.1371/journal.pone.0167374 (PMC5130269; doi:10.1371/journal.pone.0167374)

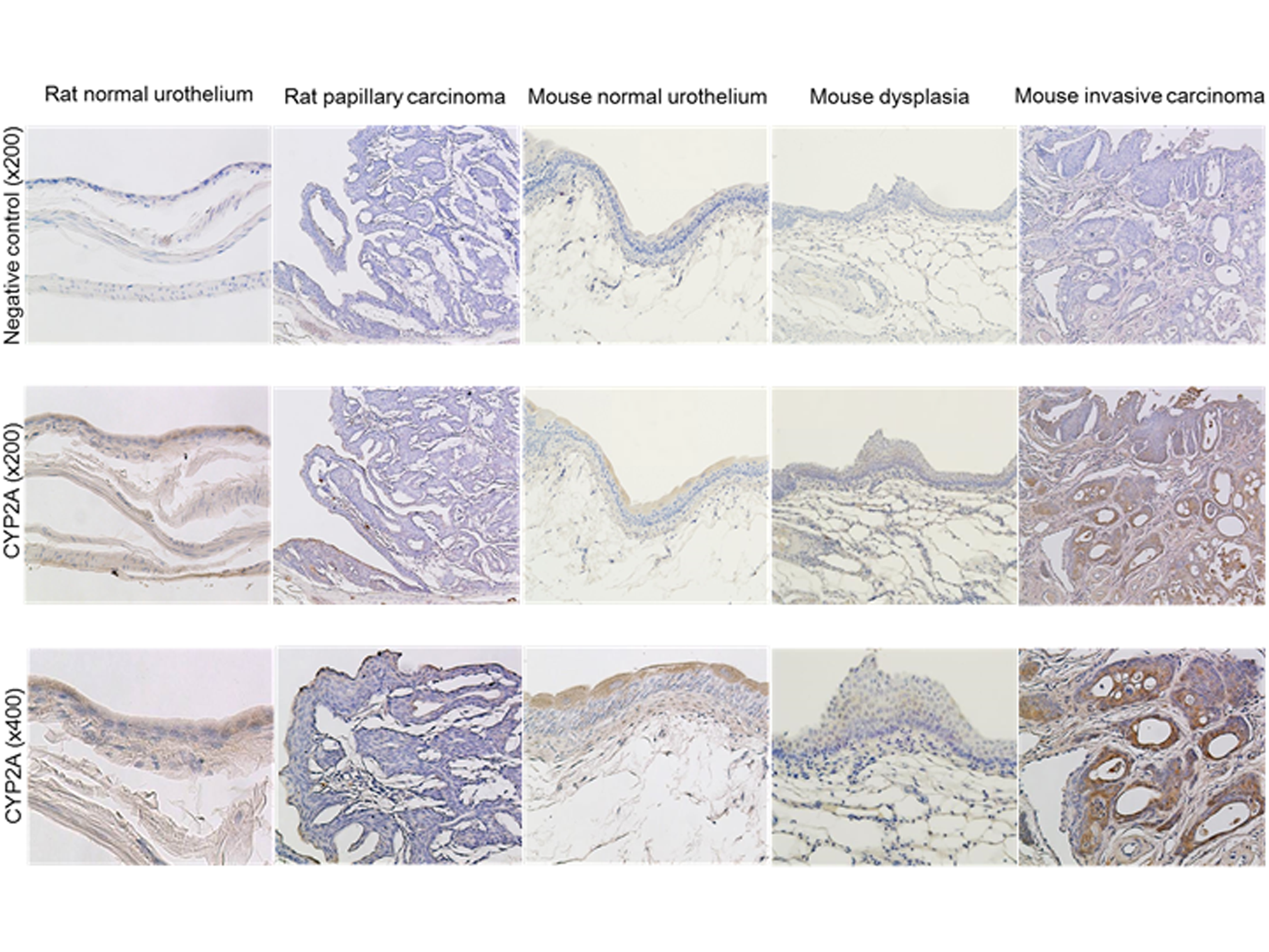

Supplement: S1 Fig — (TIF) [file pone.0167374.s001.tif]

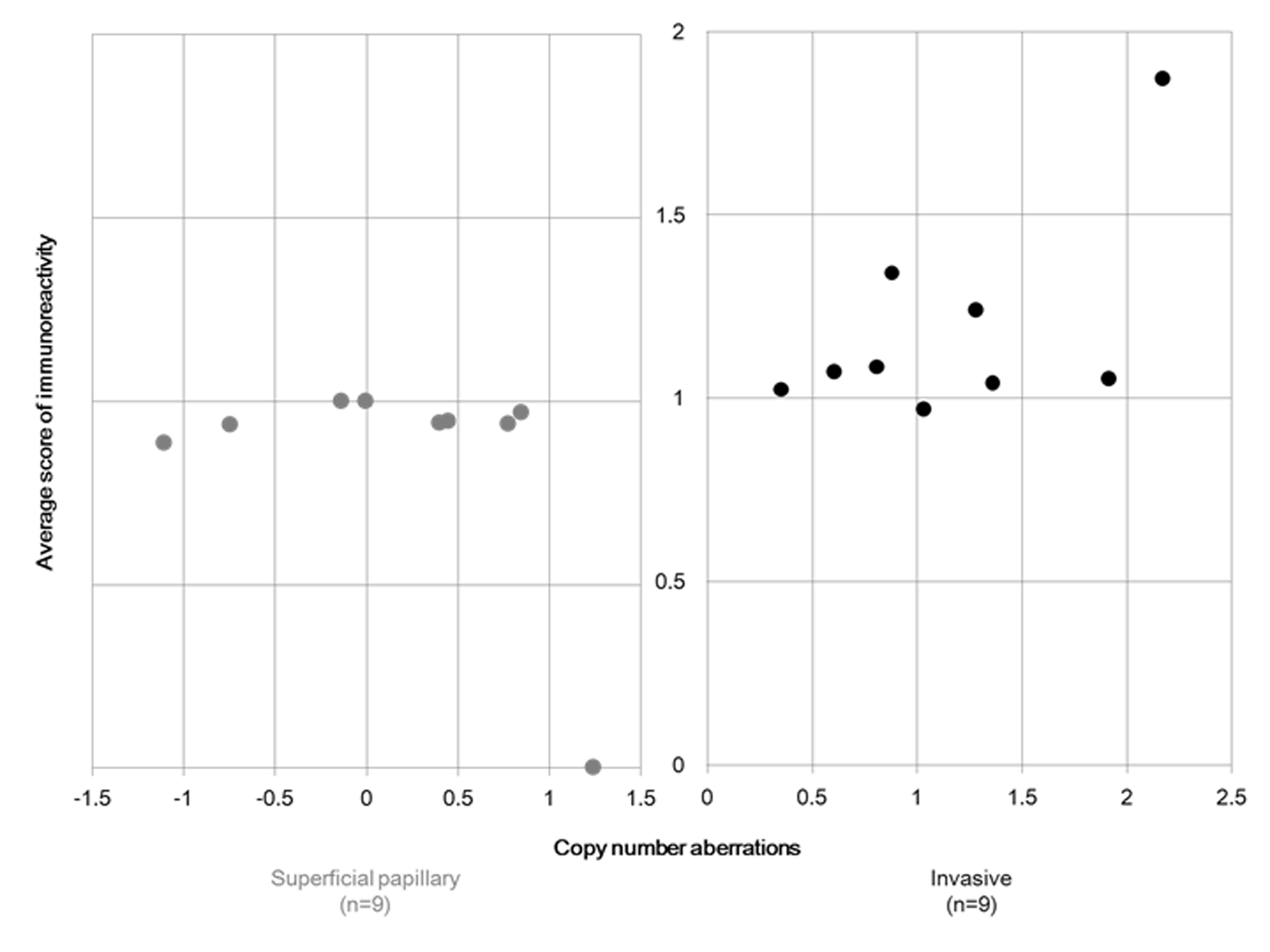

Supplement: S2 Fig — (TIF) [file pone.0167374.s002.tif]

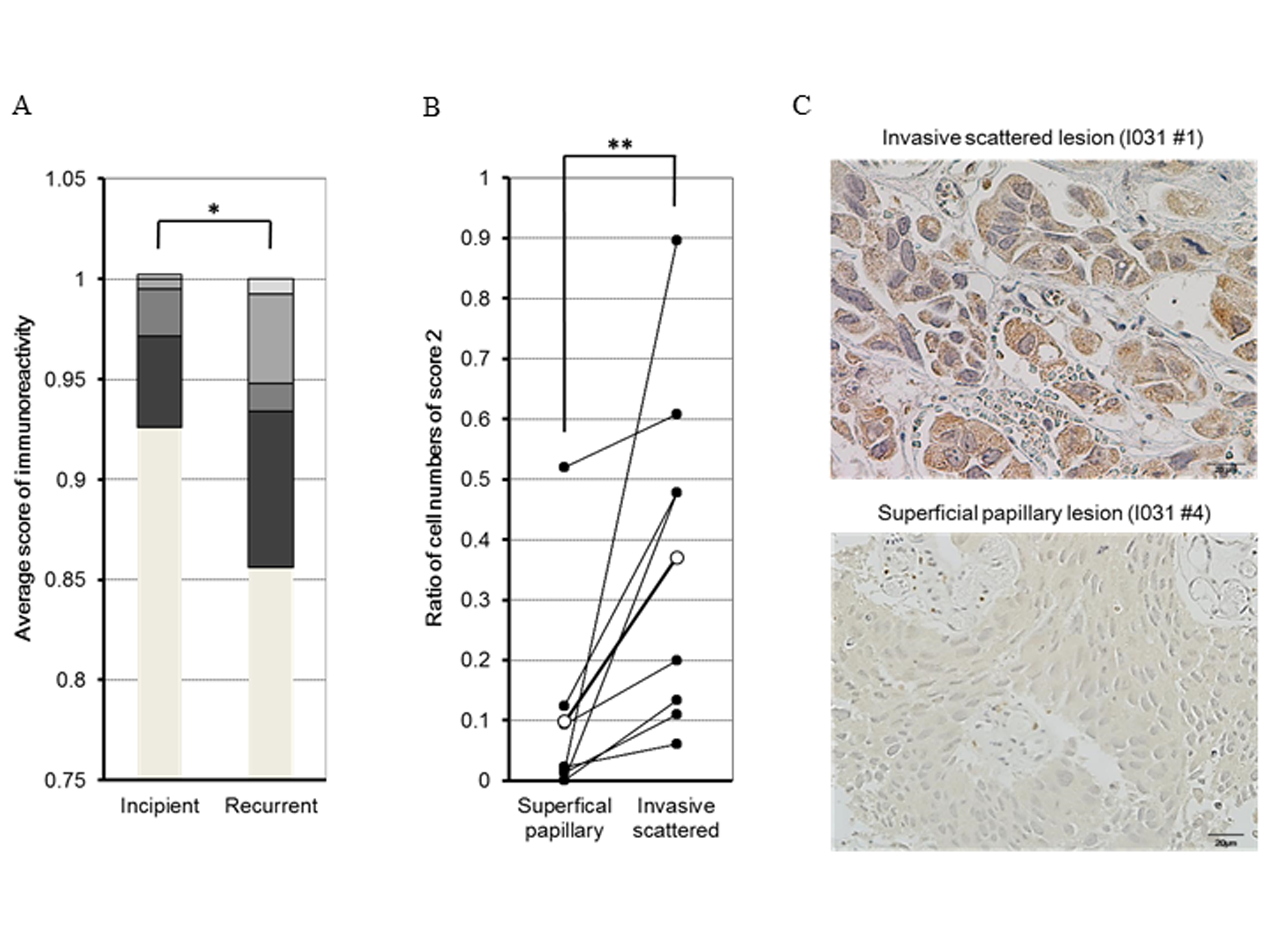

Supplement: S3 Fig — (A) CYP2A6 immunoreactivity in incipient (n = 18) and recurrent (n = 27) cases of superficial papillary tumors. The mean value based on the immunohistochemical score and cell number ratio in each TUR sample is shown. *p = 0.196 (incipient versus recurrent cases; Mann-Whitney U-test, 95th, 75th, 50th, 25th, and 10th percentile). (B) CYP2A6 immunoreactivity in 8 paired samples of superficial papillary and invasive scattered lesions derived from the same patient. The ratio of cells with score 2 in the same case is shown. Black and white circles represent a data plot and a mean plot, respectively. **p = 0.0162 (superficial papillary versus invasive scattered lesions; paired t-test). (C) CYP2A6 immunoreactivity in a representative case demonstrating superficial papillary and invasive scattered lesions. (TIF) [file pone.0167374.s003.tif]
