# Supplementary material for: Genomic Landscape of Experimental Bladder Cancer in Rodents and Its Application to Human Bladder Cancer: Gene Amplification and Potential Overexpression of Cyp2a5/CYP2A6 Are Associated with the Invasive Phenotype
Source: PLoS One. 2016 Nov 30;11(11):e0167374. doi: 10.1371/journal.pone.0167374 (PMC5130269; doi:10.1371/journal.pone.0167374)
Supplement: S1 Table — (DOCX) [file pone.0167374.s004.docx]

**Supporting Table 1. Primer Sequences for qPCR Analysis of Mouse and Human Primary Bladder Tumors.**

| **Species** | **Gene** | **Forward primer** | **Reverse primer** | **RefSeq ID** |
| --- | --- | --- | --- | --- |
| Mouse | *Olfr1184* | GCGGAACATTGAGGTGTTTT | CAGTTTGGGTGTCACACAGG | NC_000068.7  NM_146823.1  NP_667034.1 |
| Mouse | *Rapsn* | GGTCCGTTCCTACCACTCAA | CATGTGGTACTGGCTCATGG | NC_000068.7  NM_009023.3  NP_033049.2 |
| Mouse | *Cyp2g1* | GGCAGAATCCCCTTTTTCTC | CCTGTGACCTCAGACAGCAA | NC_000073.6  NM_013809.1  NP_038837.1 |
| Mouse | *Cyp2a5* | GGGGGACAGTGGACTACTCA | AACCTTGCCCAAGAAATGTG | NC_000073.6  NM_007812.4  NP_031838.2 |
| Mouse | *Cyp2a22* | TGCTCTTCCCTCTCTCCTCT | TCTCCCTCCCTCTTGATCCA | NC_000073.6  NM_001101467.1  NP_001094937.1 |
| Mouse | *Cyp2a12* | GTCCCTCAGGCTAAGTGCAG | GCCTCTTGATGCTGGTTAGC | NC_000073.6  NM_133657.1  NP_598418.1 |
| Mouse | *Rab4b* | CATGGTCCCTGATGAGTGTG | GTGGTGCTGGGACTGTACCT | NC_000073.6  NM_029391.2  NP_083667.1 |
| Mouse | *Klhl18* | TGTGGGAGCTCAGCTATCCT | GCTCCTCAGTGGGTTCTCTG | NC_000075.6  NM_177771.5  NP_808439.2 |
| Mouse | *Kif9* | CAGGAAGGCAAGAGGATCAG | GCCACCTTCTTGCTTCAGTC | NC_000075.6  variant (2)  NM_010628.3  NP_034758.2  variant (1)  NM_001163569.1  NP_001157041.1 |
| Mouse | *Nradd* | CTGGACTCTGAGGCAGTTCC | GAGGAGAGTCCACGAAGACG | NC_000075.6  NM_026012.2  NP_080288.1 |
| Mouse | *Stxbp4* | AGGTGGCCTGTACCTTCCTT | ACAACCGCCTATCCACACTC | NC_000077.6  NM_011505.2 NP_035635.1 |
| Mouse | *Cox11* | AGGATGAATGCTACCCATGC | AGAAGGCAAACTTGGAGCAA | NC_000077.6  NM_199008.2  NP_950173.1 |
| Mouse | *L1td1*  (LINE-1 type transposase domain containing 1) | AATGGCCTCGATTTTGTTTG | GGCCCTAGGTTCAGATCTCC | NC_000070.6  NM_001081202.1  NP_001074671.1 |
| Human | *RAB4B* | TAACGTCTGTCCCCCTGAAC | AATGCTTTATGGGGGTAGGG | NC_000019.9  NM_016154.4  NP_057238.3 |
| Human | *CYP2A6* | GACCAGGCTGAGGAGTTCAG | CTCCAGTTGGCAGGAGAGTC | NC_000019.9  NM_000762.5  NP_000753.3 |
| Human | *CYP2A13* | ACCTTCGACTGGCTCTTCAA | GGAGATGGGGAGAGAAGACC | NC_000019.9  NM_000766.4  NP_000757.2 |
| Human | *CYP2F1* | TCTCAATCCTGGGAAACCTG | GGAGCCATACTCCTTGCTCA | NC_000019.9  NM_000774.3  NP_000765.2 |
| Human | *Line-1*  L1RE1(LINE1 retrotransposable element 1) | AAAGCCGCTCAACTACATGG | TGCTTTGAATGCGTCCCAGAG | * |

*Supporting information for Wang *et al*. (2002) *Proc. Natl. Acad Sci. USA*, 10.1073/pnas.202610899
