# Supplementary material for: Genomic Landscape of Experimental Bladder Cancer in Rodents and Its Application to Human Bladder Cancer: Gene Amplification and Potential Overexpression of Cyp2a5/CYP2A6 Are Associated with the Invasive Phenotype
Source: PLoS One. 2016 Nov 30;11(11):e0167374. doi: 10.1371/journal.pone.0167374 (PMC5130269; doi:10.1371/journal.pone.0167374)
Supplement: S2 Table — (DOCX) [file pone.0167374.s005.docx]

**Supporting Table 2. Primer Sequences for qPCR Analysis of Human Cell Lines.**

| **Gene** | **Forward primer** | **Reverse primer** | **Probe No.** | **Position** |
| --- | --- | --- | --- | --- |
| *CYP2A6* | AATTTGAATGGGCCTGTGTC | CAGGCAGAGGGAAATCAGTC | #43 | 41352684 |
|  |  |  |  | 41352743 |
| *CYP2A7* | AAGATCCTGTCTTTCCTGGCTA | TGGACCAGAGTCTTAGGAAAGG | #89 | 41387782 |
|  |  |  |  | 41387853 |
| *CYP2B6* | CGCAGACATGTGAAGAATCAG | TCCCTGTCTCACCGTCTCTC | #85 | 41513085 |
|  |  |  |  | 41513147 |
| *CYP2A13* | CCCAACCTGCCTCATTACAC | GCCCAGCATAGGGAACACT | #37 | 41600822 |
|  |  |  |  | 41600890 |
| *CYP2F1* | GTCCTCAGCGGGTACCAA | GCGGCCACTAAACTCCTCT | #81 | 41622417 |
|  |  |  |  | 41622482 |
| *TGFB1* | TGCCAACTCACCTCTCTGACT | GCTACGAGATGCGCTTGG | #36 | 41837934 |
|  |  |  |  | 41838014 |
