# Supplementary material for: Genomic Landscape of Experimental Bladder Cancer in Rodents and Its Application to Human Bladder Cancer: Gene Amplification and Potential Overexpression of Cyp2a5/CYP2A6 Are Associated with the Invasive Phenotype
Source: PLoS One. 2016 Nov 30;11(11):e0167374. doi: 10.1371/journal.pone.0167374 (PMC5130269; doi:10.1371/journal.pone.0167374)
Supplement: S3 Table — (DOCX) [file pone.0167374.s006.docx]

**Supporting Table 3. Copy number changes investigated by qPCR.**

| Chromosome | Gene | Copy number changes | | | | | |
| --- | --- | --- | --- | --- | --- | --- | --- |
|  |  | Tumor 1 | Tumor 2 | Tumor 3 | Tumor 4 | Tumor 5 | Tumor 6 |
| 2D-E1 | *Olfr1184* | 1.8 | 2.3 | 1.5 | 1.3 | 0.8 | 2.1 |
|  | *Rapsn* | -0.4 | 0.1 | 1.7 | 1.2 | -0.1 | 0.2 |
| 7qA3 | *Cyp2g1* | -0.2 | 0.3 | 1 | 0.5 | 0.4 | -0.2 |
|  | *Cyp2a5* | 2.6 | 1.3 | 1.4 | 1.5 | 0.4 | 0.9 |
|  | *Cyp2a22* | 1.9 | 2.6 | 2.1 | 2.4 | 3.3 | 3.3 |
|  | *Cyp2a12* | 0.1 | 0.8 | 1.7 | 1 | 1.1 | 0.2 |
|  | *Rab4b* | -0.3 | -0.7 | 1.3 | 0.9 | 1.1 | 0.1 |
| 9F2 | *Klhl18* | 0.1 | 0.5 | 1 | 0.6 | 0.3 | -0.1 |
|  | *Kif9* | 0.4 | 0 | 1 | 0.7 | -0.1 | 0.2 |
|  | *Nradd* | 0.1 | 0.3 | 1.9 | 0.9 | 0.9 | -0.3 |
| 11C-D | *Stxbp4* | -0.1 | 0.3 | 0.2 | 0.7 | -0.4 | -0.1 |
|  | *Cox11* | 0.2 | 0.8 | 2.4 | 0.7 | 1.2 | 0.3 |
