# Supplementary material for: Genomic Landscape of Experimental Bladder Cancer in Rodents and Its Application to Human Bladder Cancer: Gene Amplification and Potential Overexpression of Cyp2a5/CYP2A6 Are Associated with the Invasive Phenotype
Source: PLoS One. 2016 Nov 30;11(11):e0167374. doi: 10.1371/journal.pone.0167374 (PMC5130269; doi:10.1371/journal.pone.0167374)
Supplement: S4 Table — (DOCX) [file pone.0167374.s007.docx]

**Supporting Table 4. Immunohistochemical analysis of 104 primary bladder tumors resected by TUR.**

| Case ID | Evaluated point ID | Lesions | Number of tumor cells | | |
| --- | --- | --- | --- | --- | --- |
|  |  |  | Immunohisto- | Immunohisto- | Immunohisto- |
|  |  |  | chemical score 0 | chemical score 1 | chemical score 2 |
| S001 | #1 | Superficial papillary | 13 | 199 | 0 |
| S002 | #1 | Superficial papillary | 12 | 189 | 0 |
| S003 | #1 | Superficial papillary | 0 | 200 | 1 |
| S004 | #1 | Superficial papillary | 0 | 200 | 0 |
| S005 | #1 | Superficial papillary | 1 | 200 | 0 |
| S006 | #1 | Superficial papillary | 13 | 195 | 0 |
| S007 | #1 | Superficial papillary | 0 | 200 | 0 |
| S008 | #1 | Superficial papillary | 0 | 200 | 0 |
| S009 | #1 | Superficial papillary | 14 | 205 | 0 |
| S010 | #1 | Superficial papillary | 2 | 198 | 0 |
| S011 | #1 | Superficial papillary | 23 | 180 | 0 |
| S012 | #1 | Superficial papillary | 10 | 190 | 0 |
| S013 | #1 | Superficial papillary | 86 | 119 | 0 |
| S014 | #1 | Superficial papillary | 4 | 200 | 0 |
| S015 | #1 | Superficial papillary | 0 | 200 | 0 |
| S016 | #1 | Superficial papillary | 7 | 217 | 0 |
| S017 | #1 | Superficial papillary | 17 | 187 | 0 |
| S018 | #1 | Superficial papillary | 1 | 200 | 0 |
| S019 | #1 | Superficial papillary | 8 | 196 | 0 |
| S020 | #1 | Superficial papillary | 11 | 192 | 0 |
| S021 | #1 | Superficial papillary | 12 | 204 | 0 |
| S022 | #1 | Superficial papillary | 200 | 0 | 0 |
| S023 | #1 | Superficial papillary | 0 | 200 | 0 |
| S024 | #1 | Superficial papillary | 0 | 200 | 0 |
| S025 | #1 | Superficial papillary | 5 | 200 | 0 |
| S026 | #1 | Superficial papillary | 2 | 200 | 0 |
| S027 | #1 | Superficial papillary | 37 | 217 | 0 |
|  | #2 | Superficial papillary | 0 | 200 | 0 |
| S028 | #1 | Superficial papillary | 0 | 200 | 0 |
| S029 | #1 | Superficial papillary | 1 | 200 | 0 |
| S030 | #1 | Superficial papillary | 200 | 0 | 0 |
|  | #2 | Superficial papillary | 200 | 3 | 0 |
|  | #3 | Superficial papillary | 200 | 9 | 2 |
| S031 | #1 | Superficial papillary | 0 | 200 | 0 |
| S032 | #1 | Superficial papillary | 3 | 200 | 0 |
| S033 | #1 | Superficial papillary | 0 | 200 | 0 |
|  | #2 | Superficial papillary | 13 | 410 | 30 |
|  | #3 | Superficial papillary | 6 | 200 | 0 |
| S034 | #1 | Superficial papillary | 11 | 184 | 14 |
|  | #2 | Superficial papillary | 2 | 200 | 0 |
| S035 | #1 | Superficial papillary | 9 | 192 | 0 |
| S036 | #1 | Superficial papillary | 1 | 200 | 0 |
| S037 | #1 | Superficial papillary | 1 | 200 | 0 |
|  | #2 | Superficial papillary | 24 | 177 | 0 |
|  | #3 | Superficial papillary | 20 | 219 | 0 |
| S038 | #1 | Superficial papillary | 0 | 200 | 0 |
| S039 | #1 | Superficial papillary | 8 | 200 | 0 |
| S040 | #1 | Superficial papillary | 1 | 200 | 0 |
| S041 | #1 | Superficial papillary | 1 | 200 | 0 |
| S042 | #1 | Superficial papillary | 1 | 200 | 0 |
| S043 | #1 | Superficial papillary | 18 | 193 | 0 |
| S044 | #1 | Superficial papillary | 0 | 200 | 0 |
| S045 | #1 | Superficial papillary | 1 | 200 | 0 |
| I001 | #1 | Invasive aggregated | 30 | 197 | 0 |
|  | #2 | Invasive aggregated | 22 | 223 | 4 |
|  | #3 | Invasive aggregated | 17 | 217 | 2 |
| I002 | #1 | Invasive scattered | 30 | 266 | 2 |
|  | #2 | Invasive scattered | 10 | 192 | 0 |
|  | #3 | Invasive scattered | 17 | 210 | 0 |
|  | #4 | Invasive aggregated | 16 | 266 | 1 |
|  | #5 | Invasive aggregated | 17 | 212 | 4 |
|  | #6 | Invasive aggregated | 24 | 213 | 4 |
| I003 | #1 | Invasive scattered | 130 | 57 | 42 |
|  | #2 | Invasive scattered | 85 | 74 | 45 |
|  | #3 | Invasive scattered | 142 | 120 | 110 |
|  | #4 | Invasive scattered | 9 | 200 | 5 |
|  | #5 | Invasive scattered | 53 | 120 | 27 |
|  | #6 | Invasive scattered | 41 | 180 | 0 |
|  | #7 | Invasive scattered | 23 | 177 | 11 |
|  | #8 | Invasive scattered | 37 | 159 | 7 |
|  | #9 | Invasive scattered | 82 | 141 | 34 |
| I004 | #1 | Invasive scattered | 4 | 320 | 10 |
|  | #2 | Invasive scattered | 11 | 225 | 2 |
|  | #3 | Invasive scattered | 25 | 180 | 18 |
| I005 | #1 | Invasive scattered | 24 | 236 | 0 |
|  | #2 | Invasive scattered | 43 | 193 | 2 |
|  | #3 | Invasive scattered | 59 | 187 | 0 |
| I006 | #1 | Invasive aggregated | 15 | 187 | 0 |
|  | #2 | Invasive aggregated | 7 | 279 | 0 |
|  | #3 | Invasive aggregated | 16 | 326 | 0 |
| I007 | #1 | Invasive scattered | 25 | 186 | 0 |
|  | #2 | Invasive scattered | 34 | 234 | 0 |
|  | #3 | Invasive scattered | 29 | 119 | 74 |
| I008 | #1 | Invasive aggregated | 5 | 376 | 0 |
|  | #2 | Invasive aggregated | 45 | 374 | 15 |
|  | #3 | Invasive aggregated | 2 | 297 | 0 |
| I009 | #1 | Invasive scattered | 18 | 345 | 3 |
|  | #2 | Invasive scattered | 21 | 186 | 2 |
|  | #3 | Invasive scattered | 20 | 197 | 2 |
| I010 | #1 | Invasive scattered | 7 | 107 | 172 |
|  | #2 | Invasive scattered | 20 | 147 | 175 |
|  | #3 | Invasive scattered | 10 | 497 | 8 |
| I011 | #1 | Invasive aggregated | 4 | 343 | 18 |
|  | #2 | Invasive aggregated | 2 | 369 | 38 |
|  | #3 | Invasive aggregated | 7 | 222 | 7 |
| I012 | #1 | Invasive scattered | 5 | 526 | 0 |
|  | #2 | Invasive aggregated | 5 | 479 | 0 |
|  | #3 | Invasive scattered | 4 | 477 | 0 |
| I013 | #1 | Invasive scattered | 29 | 437 | 3 |
|  | #2 | Invasive scattered | 9 | 429 | 7 |
|  | #3 | Invasive scattered | 13 | 535 | 3 |
|  | #4 | Invasive scattered | 27 | 67 | 107 |
|  | #5 | Invasive scattered | 23 | 71 | 107 |
|  | #6 | Invasive scattered | 13 | 97 | 97 |
| I014 | #1 | Invasive scattered | 5 | 334 | 0 |
|  | #2 | Invasive scattered | 11 | 263 | 0 |
|  | #3 | Invasive scattered | 8 | 379 | 0 |
| I015 | #1 | Invasive scattered | 10 | 500 | 40 |
|  | #2 | Invasive scattered | 36 | 112 | 184 |
|  | #3 | Invasive scattered | 16 | 342 | 53 |
| I016 | #1 | Invasive scattered | 40 | 270 | 82 |
|  | #2 | Invasive scattered | 11 | 168 | 21 |
|  | #3 | Invasive scattered | 12 | 198 | 2 |
| I017 | #1 | Invasive scattered | 7 | 305 | 7 |
|  | #2 | Invasive scattered | 15 | 318 | 20 |
|  | #3 | Invasive scattered | 14 | 304 | 3 |
| I018 | #1 | Invasive scattered | 15 | 514 | 18 |
|  | #2 | Invasive scattered | 8 | 465 | 2 |
|  | #3 | Invasive scattered | 15 | 623 | 14 |
| I019 | #1 | Invasive aggregated | 2 | 402 | 6 |
|  | #2 | Invasive aggregated | 4 | 321 | 0 |
|  | #3 | Invasive aggregated | 1 | 408 | 0 |
| I020 | #1 | Invasive aggregated | 4 | 502 | 1 |
|  | #2 | Invasive aggregated | 3 | 327 | 27 |
|  | #3 | Invasive scattered | 3 | 82 | 176 |
| I021 | #1 | Invasive scattered | 9 | 60 | 231 |
|  | #2 | Invasive scattered | 0 | 219 | 11 |
|  | #3 | Invasive scattered | 3 | 147 | 159 |
| I022 | #1 | Invasive aggregated | 16 | 88 | 173 |
|  | #2 | Invasive aggregated | 5 | 107 | 137 |
|  | #3 | Invasive aggregated | 19 | 151 | 64 |
| I023 | #1 | Invasive aggregated | 7 | 404 | 53 |
|  | #2 | Invasive aggregated | 13 | 288 | 52 |
|  | #3 | Invasive aggregated | 13 | 165 | 46 |
| I024 | #1 | Invasive aggregated | 15 | 212 | 26 |
|  | #2 | Invasive aggregated | 16 | 337 | 24 |
|  | #3 | Invasive scattered | 13 | 256 | 64 |
|  | #4 | Invasive aggregated | 17 | 445 | 7 |
|  | #5 | Invasive aggregated | 9 | 360 | 24 |
|  | #6 | Invasive aggregated | 13 | 320 | 11 |
|  | #7 | Invasive scattered | 8 | 320 | 69 |
| I025 | #1 | Invasive scattered | 50 | 203 | 78 |
|  | #2 | Invasive scattered | 42 | 279 | 98 |
|  | #3 | Invasive scattered | 32 | 389 | 47 |
|  | #4 | Invasive scattered | 24 | 203 | 97 |
| I026 | #1 | Invasive aggregated | 19 | 359 | 3 |
|  | #2 | Invasive aggregated | 2 | 200 | 2 |
|  | #3 | Invasive aggregated | 15 | 334 | 19 |
|  | #4 | Invasive aggregated | 60 | 213 | 25 |
| I027 | #1 | Invasive aggregated | 19 | 332 | 3 |
|  | #2 | Invasive aggregated | 4 | 213 | 6 |
|  | #3 | Invasive aggregated | 1 | 300 | 6 |
|  | #4 | Invasive scattered | 28 | 247 | 41 |
|  | #5 | Invasive scattered | 15 | 209 | 25 |
|  | #6 | Invasive scattered | 10 | 229 | 29 |
| I028 | #1 | Invasive aggregated | 9 | 195 | 2 |
|  | #2 | Invasive aggregated | 8 | 295 | 0 |
|  | #3 | Invasive aggregated | 4 | 200 | 0 |
| I029 | #1 | Invasive scattered | 10 | 244 | 76 |
|  | #2 | Invasive scattered | 13 | 193 | 26 |
|  | #3 | Invasive scattered | 28 | 186 | 40 |
|  | #4 | Invasive scattered | 18 | 139 | 59 |
| I030 | #1 | Invasive scattered | 11 | 58 | 194 |
|  | #2 | Invasive scattered | 5 | 35 | 167 |
|  | #3 | Invasive scattered | 4 | 12 | 370 |
|  | #4 | Invasive aggregated | 1 | 220 | 11 |
|  | #5 | Invasive aggregated | 7 | 355 | 5 |
|  | #6 | Invasive aggregated | 3 | 578 | 14 |
|  | #7 | Invasive aggregated | 1 | 200 | 0 |
|  | #1 | Invasive scattered | 0 | 27 | 214 |
| I031 | #2 | Invasive scattered | 16 | 22 | 289 |
|  | #3 | Invasive scattered | 7 | 17 | 264 |
| I032 | #1 | Invasive aggregated | 0 | 319 | 5 |
|  | #2 | Invasive aggregated | 5 | 417 | 7 |
|  | #3 | Invasive aggregated | 6 | 239 | 65 |
|  | #4 | Invasive scattered | 0 | 30 | 209 |
|  | #5 | Invasive scattered | 2 | 65 | 221 |
|  | #6 | Invasive scattered | 4 | 50 | 296 |
| I033 | #1 | Invasive aggregated | 0 | 200 | 6 |
|  | #2 | Invasive aggregated | 0 | 200 | 6 |
|  | #3 | Invasive aggregated | 11 | 446 | 11 |
|  | #4 | Invasive scattered | 19 | 292 | 24 |
|  | #5 | Invasive scattered | 2 | 142 | 212 |
|  | #6 | Invasive scattered | 19 | 42 | 237 |
| I034 | #1 | Invasive aggregated | 22 | 366 | 58 |
|  | #2 | Invasive aggregated | 8 | 239 | 2 |
|  | #3 | Invasive aggregated | 4 | 277 | 9 |
|  | #4 | Invasive scattered | 13 | 165 | 46 |
|  | #5 | Invasive scattered | 3 | 341 | 15 |
|  | #6 | Invasive scattered | 4 | 244 | 20 |
|  | #7 | Invasive scattered | 1 | 212 | 7 |
| I035 | #1 | Invasive aggregated | 0 | 320 | 104 |
|  | #2 | Invasive aggregated | 0 | 354 | 146 |
|  | #3 | Invasive aggregated | 1 | 431 | 21 |
|  | #4 | Invasive scattered | 11 | 200 | 205 |
|  | #5 | Invasive scattered | 0 | 5 | 203 |
|  | #6 | Invasive scattered | 4 | 133 | 168 |
|  | #7 | Invasive scattered | 8 | 135 | 192 |
| I036 | #1 | Invasive scattered | 15 | 184 | 353 |
|  | #2 | Invasive scattered | 10 | 84 | 230 |
|  | #3 | Invasive scattered | 17 | 60 | 165 |
| I037 | #1 | Invasive aggregated | 4 | 249 | 19 |
|  | #2 | Invasive aggregated | 5 | 111 | 211 |
|  | #3 | Invasive aggregated | 0 | 200 | 0 |
| I038 | #1 | Invasive scattered | 25 | 176 | 62 |
|  | #2 | Invasive scattered | 18 | 166 | 37 |
|  | #3 | Invasive scattered | 10 | 278 | 8 |
| I039 | #1 | Invasive aggregated | 57 | 112 | 87 |
|  | #2 | Invasive aggregated | 91 | 102 | 106 |
|  | #3 | Invasive aggregated | 44 | 172 | 50 |
| I040 | #1 | Invasive aggregated | 18 | 537 | 184 |
|  | #2 | Invasive aggregated | 18 | 464 | 74 |
|  | #3 | Invasive aggregated | 19 | 540 | 58 |
| I041 | #1 | Invasive aggregated | 4 | 370 | 2 |
|  | #2 | Invasive aggregated | 3 | 327 | 1 |
|  | #3 | Invasive aggregated | 3 | 381 | 33 |
| I042 | #1 | Invasive aggregated | 14 | 306 | 21 |
|  | #2 | Invasive aggregated | 4 | 618 | 9 |
|  | #3 | Invasive aggregated | 1 | 312 | 10 |
| I043 | #1 | Invasive aggregated | 6 | 224 | 94 |
|  | #2 | Invasive aggregated | 18 | 312 | 44 |
|  | #3 | Invasive aggregated | 14 | 290 | 52 |
| I044 | #1 | Invasive aggregated | 4 | 352 | 2 |
|  | #2 | Invasive aggregated | 11 | 602 | 19 |
|  | #3 | Invasive aggregated | 2 | 200 | 4 |
|  | #4 | Invasive scattered | 3 | 165 | 81 |
|  | #5 | Invasive scattered | 5 | 217 | 70 |
|  | #6 | Invasive aggregated | 14 | 263 | 4 |
| I045 | #1 | Invasive scattered | 23 | 95 | 129 |
|  | #2 | Invasive scattered | 41 | 153 | 160 |
|  | #3 | Invasive scattered | 22 | 176 | 15 |
|  | #4 | Invasive scattered | 27 | 120 | 64 |
|  | #5 | Invasive scattered | 38 | 150 | 68 |
|  | #6 | Invasive scattered | 74 | 110 | 58 |
|  | #7 | Invasive scattered | 70 | 124 | 78 |
| I046 | #1 | Invasive aggregated | 4 | 136 | 76 |
|  | #2 | Invasive aggregated | 10 | 154 | 71 |
|  | #3 | Invasive aggregated | 24 | 108 | 87 |
|  | #4 | Invasive scattered | 6 | 284 | 72 |
| I047 | #1 | Invasive aggregated | 23 | 182 | 9 |
|  | #2 | Invasive aggregated | 30 | 164 | 12 |
|  | #3 | Invasive scattered | 6 | 156 | 42 |
| I048 | #1 | Invasive aggregated | 6 | 207 | 3 |
|  | #2 | Invasive aggregated | 23 | 267 | 116 |
|  | #3 | Invasive aggregated | 32 | 347 | 17 |
|  | #4 | Invasive aggregated | 7 | 124 | 72 |
|  | #5 | Invasive aggregated | 9 | 217 | 60 |
|  | #6 | Invasive aggregated | 34 | 161 | 64 |
|  | #7 | Invasive aggregated | 7 | 390 | 12 |
| I049 | #1 | Invasive scattered | 18 | 204 | 74 |
|  | #2 | Invasive scattered | 32 | 218 | 100 |
|  | #3 | Invasive scattered | 28 | 194 | 76 |
|  | #4 | Invasive scattered | 40 | 276 | 52 |
| I050 | #1 | Invasive scattered | 6 | 368 | 13 |
|  | #2 | Invasive scattered | 10 | 389 | 22 |
|  | #3 | Invasive scattered | 8 | 233 | 26 |
|  | #4 | Invasive scattered | 9 | 146 | 121 |
| I051 | #1 | Invasive aggregated | 14 | 458 | 0 |
|  | #2 | Invasive aggregated | 0 | 200 | 0 |
|  | #3 | Invasive aggregated | 0 | 403 | 16 |
| I052 | #1 | Invasive aggregated | 1 | 344 | 4 |
|  | #2 | Invasive aggregated | 0 | 249 | 10 |
|  | #3 | Invasive aggregated | 0 | 200 | 0 |
|  | #4 | Invasive scattered | 7 | 211 | 27 |
| I053 | #1 | Invasive aggregated | 11 | 514 | 21 |
|  | #2 | Invasive aggregated | 18 | 341 | 29 |
|  | #3 | Invasive aggregated | 7 | 226 | 22 |
| I054 | #1 | Invasive aggregated | 10 | 167 | 30 |
|  | #2 | Invasive aggregated | 9 | 287 | 14 |
|  | #3 | Invasive aggregated | 6 | 267 | 38 |
|  | #4 | Invasive aggregated | 0 | 330 | 20 |
| I055 | #1 | Invasive aggregated | 4 | 248 | 30 |
|  | #2 | Invasive scattered | 5 | 145 | 75 |
|  | #3 | Invasive scattered | 11 | 156 | 66 |
|  | #4 | Invasive aggregated | 1 | 249 | 14 |
|  | #5 | Invasive scattered | 11 | 101 | 125 |
|  | #6 | Invasive scattered | 10 | 126 | 98 |
|  | #7 | Invasive scattered | 8 | 312 | 62 |
|  | #8 | Invasive scattered | 0 | 151 | 81 |
|  | #9 | Invasive aggregated | 0 | 197 | 18 |
| I056 | #1 | Invasive scattered | 42 | 130 | 142 |
|  | #2 | Invasive scattered | 9 | 128 | 139 |
|  | #3 | Invasive scattered | 8 | 196 | 174 |
|  | #4 | Invasive scattered | 28 | 72 | 152 |
| I057 | #1 | Invasive scattered | 10 | 268 | 96 |
|  | #2 | Invasive scattered | 9 | 61 | 331 |
|  | #3 | Invasive scattered | 11 | 52 | 155 |
| I058 | #1 | Invasive scattered | 5 | 263 | 10 |
|  | #2 | Invasive scattered | 1 | 467 | 24 |
|  | #3 | Invasive scattered | 3 | 287 | 3 |
| I059 | #1 | Invasive scattered | 3 | 169 | 93 |
|  | #2 | Invasive scattered | 7 | 259 | 32 |
|  | #3 | Invasive scattered | 10 | 134 | 125 |
