# Supplementary material for: Genomic Landscape of Experimental Bladder Cancer in Rodents and Its Application to Human Bladder Cancer: Gene Amplification and Potential Overexpression of Cyp2a5/CYP2A6 Are Associated with the Invasive Phenotype
Source: PLoS One. 2016 Nov 30;11(11):e0167374. doi: 10.1371/journal.pone.0167374 (PMC5130269; doi:10.1371/journal.pone.0167374)
Supplement: S5 Table — (DOCX) [file pone.0167374.s008.docx]

**Supporting Table 5. CYP2A6 expression in superficial papillary lesions found among TUR samples of invasive scattered lesions present in the same patient.**

| Case ID | Evaluated point ID | Lesions | Number of tumor cells | | |
| --- | --- | --- | --- | --- | --- |
|  |  |  | Immunohisto- | Immunohisto- | Immunohisto- |
|  |  |  | chemical score 0 | chemical score 1 | chemical score 2 |
| I003 | #10 | Superficial papillary | 200 | 0 | 0 |
|  | #11 | Superficial papillary | 200 | 0 | 0 |
|  | #12 | Superficial papillary | 200 | 0 | 0 |
| I021 | #4 | Superficial papillary | 5 | 399 | 8 |
|  | #5 | Superficial papillary | 8 | 338 | 74 |
|  | #6 | Superficial papillary | 0 | 404 | 80 |
| I031 | #4 | Superficial papillary | 6 | 236 | 0 |
|  | #5 | Superficial papillary | 15 | 362 | 12 |
|  | #6 | Superficial papillary | 22 | 286 | 3 |
| I033 | #7 | Superficial papillary | 12 | 200 | 0 |
|  | #8 | Superficial papillary | 10 | 200 | 0 |
|  | #9 | Superficial papillary | 3 | 200 | 0 |
| I035 | #8 | Superficial papillary | 16 | 212 | 170 |
|  | #9 | Superficial papillary | 10 | 395 | 144 |
|  | #10 | Superficial papillary | 6 | 32 | 411 |
| I046 | #5 | Superficial papillary | 8 | 262 | 16 |
|  | #6 | Superficial papillary | 14 | 161 | 20 |
|  | #7 | Superficial papillary | 5 | 398 | 41 |
|  | #8 | Superficial papillary | 4 | 248 | 35 |
| I052 | #5 | Superficial papillary | 5 | 200 | 3 |
|  | #6 | Superficial papillary | 6 | 200 | 4 |
|  | #7 | Superficial papillary | 1 | 200 | 1 |
| I058 | #4 | Superficial papillary | 2 | 200 | 4 |
|  | #5 | Superficial papillary | 5 | 200 | 6 |
|  | #6 | Superficial papillary | 4 | 200 | 4 |
